# Tuberculosis control interventions targeted to previously treated people in a high-incidence setting: a modelling study

**DOI:** 10.1016/S2214-109X(18)30022-6

**Published:** 2018-02-19

**Authors:** Florian M Marx, Reza Yaesoubi, Nicolas A Menzies, Joshua A Salomon, Alyssa Bilinski, Nulda Beyers, Ted Cohen

**Affiliations:** Department of Epidemiology of Microbial Diseases (F M Marx MD, T Cohen MD), Department of Health Policy and Management (R Yaesoubi PhD), Yale School of Public Health, New Haven, CT, USA; Division of Global Health Equity, Brigham and Women’s Hospital and Harvard Medical School, Boston, MA, USA (F M Marx); Desmond Tutu TB Centre, Department of Paediatrics and Child Health, Faculty of Medicine and Health Sciences, Stellenbosch University, Cape Town, South Africa (F M Marx, N Beyers PhD); Department of Global Health and Population, Harvard T H Chan School of Public Health, Boston, MA, USA (N A Menzies PhD, J A Salomon PhD); and Interfaculty Initiative in Health Policy, Harvard University, Cambridge, MA, USA (A Bilinski)

## Abstract

**Background:**

In high-incidence settings, recurrent disease among previously treated individuals contributes substantially to the burden of incident and prevalent tuberculosis. The extent to which interventions targeted to this high-risk group can improve tuberculosis control has not been established. We aimed to project the population-level effect of control interventions targeted to individuals with a history of previous tuberculosis treatment in a high-incidence setting.

**Methods:**

We developed a transmission-dynamic model of tuberculosis and HIV in a high-incidence setting with a population of roughly 40 000 people in suburban Cape Town, South Africa. The model was calibrated to data describing local demography, TB and HIV prevalence, TB case notifications and treatment outcomes using a Bayesian calibration approach. We projected the effect of annual targeted active case finding in all individuals who had previously completed tuberculosis treatment and targeted active case finding combined with lifelong secondary isoniazid preventive therapy. We estimated the effect of these targeted interventions on local tuberculosis incidence, prevalence, and mortality over a 10 year period (2016–25).

**Findings:**

We projected that, under current control efforts in this setting, the tuberculosis epidemic will remain in slow decline for at least the next decade. Additional interventions targeted to previously treated people could greatly accelerate these declines. We projected that annual targeted active case finding combined with secondary isoniazid preventive therapy in those who previously completed tuberculosis treatment would avert 40% (95% uncertainty interval [UI] 21–56) of incident tuberculosis cases and 41% (16–55) of tuberculosis deaths occurring between 2016 and 2025.

**Interpretation:**

In this high-incidence setting, the use of targeted active case finding in combination with secondary isoniazid preventive therapy in previously treated individuals could accelerate decreases in tuberculosis morbidity and mortality. Studies to measure cost and resource implications are needed to establish the feasibility of this type of targeted approach for improving tuberculosis control in settings with high tuberculosis and HIV prevalence.

**Funding:**

National Institutes of Health, German Research Foundation.

## Introduction

Worldwide, an estimated 10·4 million people developed tuberculosis and 1·8 million deaths were attributable to the disease in 2015.^1^ Substantial innovation in tuberculosis control is needed to reach the targets of the new global End TB Strategy, which aims to eliminate the disease by the year 2035.^2^ The rates of tuberculosis decline must accelerate in settings with the highest disease incidence, some of which are located in southern Africa and are facing the dual burden of tuberculosis and HIV.^3^ In these settings, the prevalence of untreated tuberculosis remains high, and conventional control approaches that rely on passive case finding can fail to identify infectious cases early enough to prevent transmission.^4–6^

Active case finding and wide-scale use of preventive therapy have been considered as enhanced activities for improving tuberculosis control, but these approaches require substantial investment.^7^ Furthermore, disappointing results from community-randomised trials of population-wide case finding and preventive therapy interventions^8,9^ have tempered enthusiasm for untargeted use of these interventions. It remains unknown whether targeting of case finding and preventive therapy to high-risk groups could be an effective approach for disease control in communities. The broader effect of a targeted approach depends on whether it is possible to prevent disease or reduce the duration of infectiousness among an easily identifiable subgroup that experiences a high relative risk of disease and is responsible for a substantial proportion of transmission.

One subgroup that might be attractive for targeted interventions is individuals with a history of previous tuberculosis treatment.^10^ Studies from southern Africa show a high incidence of recurrent tuberculosis even after previous successful treatment,^11–14^ resulting from both endogenous reactivation and exogenous reinfection.^15^ We recently documented a large burden of prevalent tuberculosis in previously treated adults in 24 high tuberculosis burden communities in southern Africa, consistent with the hypothesis that this risk group drives a substantial proportion of transmission in these settings.^16^

In this study, we used a transmission-dynamic model to project the effect of two targeted control interventions— targeted active case finding and secondary isoniazid preventive therapy—in individuals who previously completed tuberculosis treatment in a high-incidence setting in suburban Cape Town, South Africa. We estimated the effect of these targeted interventions on tuberculosis incidence, prevalence, and mortality over a 10-year period.

## Methods

### Modelling approach

**Figure 1:**
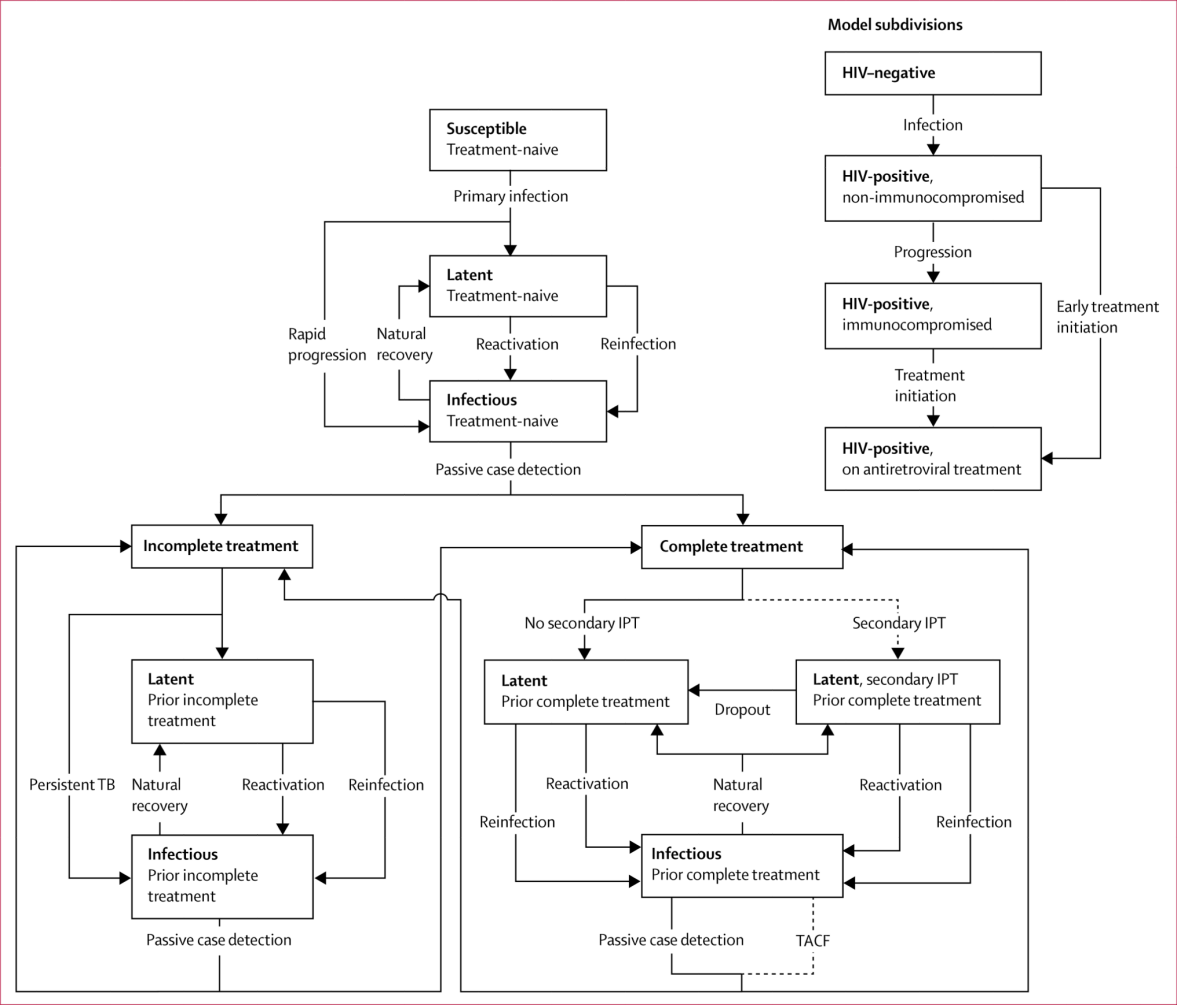
Structure of the mathematical model Dashed arrows are modelled interventions, 2°IPT=secondary isoniazid preventive therapy. TACF=targeted active case finding. Mortality rates are not shown. The childhood subcomponent and corresponding transitions are shown in the appendix.

We developed a stochastic compartmental transmission-dynamic model of the tuberculosis and HIV epidemic in a high-incidence setting of roughly 40 000 residents in suburban Cape Town, South Africa; the appendix provides details about the study setting. The tuberculosis component of our model followed the conventions of earlier models,^17–21^ with additional structure to distinguish between individuals who were never treated for tuberculosis (treatment-naive) and those who were previously treated for tuberculosis (treatment-experienced; figure 1).

We adopted previous ranges for parameters that allowed for differential partial immunity against re-infection and differential reactivation rates in treatment-experienced and treatment-naive, latently infected individuals, and differential delay in detecting tuberculosis in individuals with and without history of tuberculosis treatment (table 1). We also allowed for higher infectiousness in treatment-experienced compared with treatment-naive tuberculosis cases, as suggested by local tuberculosis prevalence surveys that reported that treatment-experienced individuals with tuberculosis were more likely to report cough and more likely to be smear-positive than treatment-naive individuals without the disease.^16^ Among individuals with incomplete tuberculosis treatment, we assumed that up to 20% remained infectious, consistent with findings from a retrospective cohort study done in the study setting.^26^ Table 1 shows a list of key model parameters describing differences in treatment-naive and treatment-experienced individuals.

The HIV component of the model accounts for HIV infection, progression to a state of immunocompromised HIV infection, and antiretroviral treatment (ART; figure 1). We also implemented a model subcomponent for children aged 0–14 years. Additional model details including the subcomponent for children are described in the appendix.

### Model initialisation and parameter estimation approach

**Figure 2:**
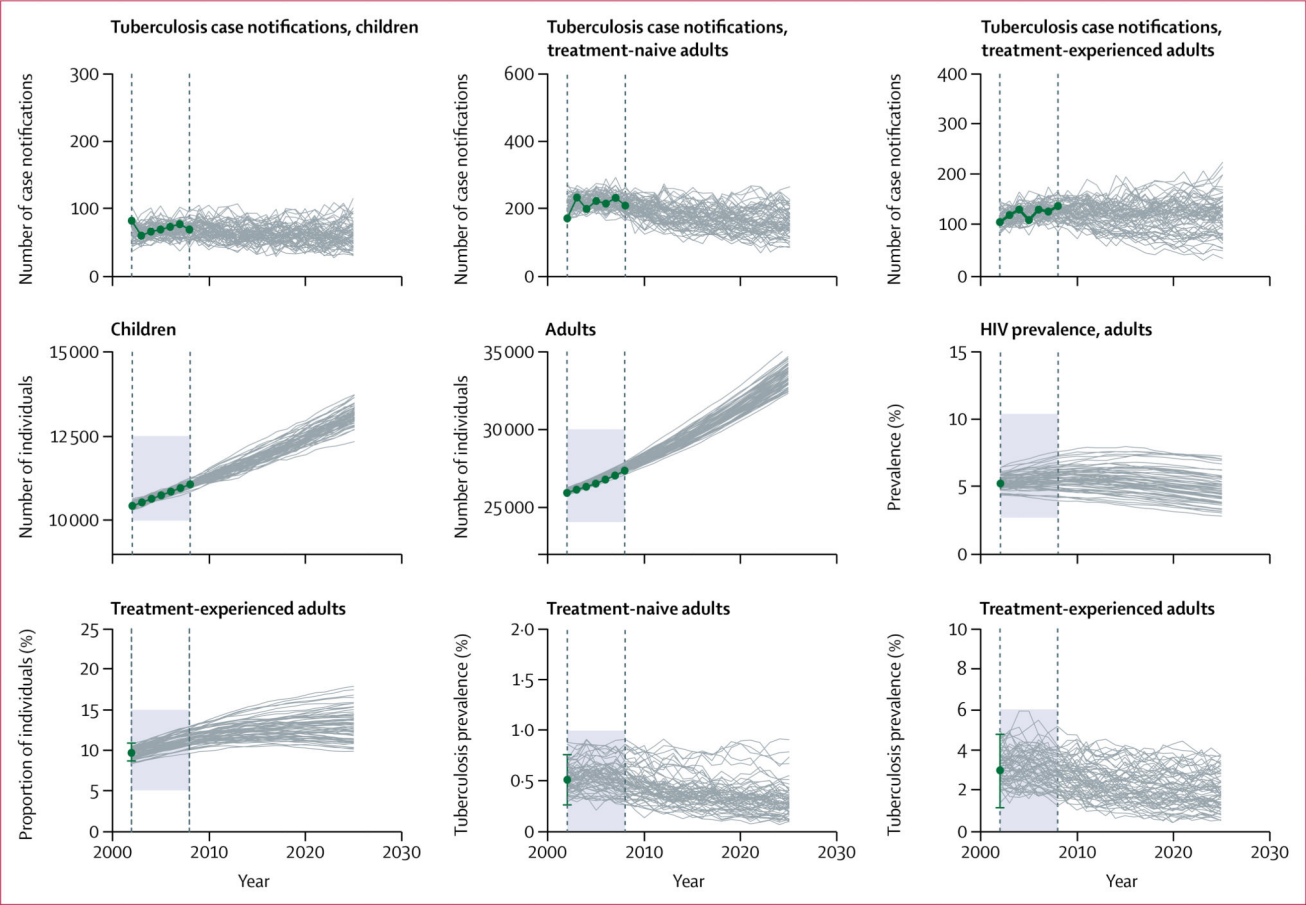
Calibration targets and fitted model trajectories Green dots denote the nine calibration targets, with error bars representing 95% CIs where applicable; grey lines represent 100 simulated trajectories produced by the calibrated model; the simulated trajectories that fell outside the feasible regions (shaded areas) were considered extremely unlikely and were eliminated by the calibration method. The interval between the dashed vertical lines shows the model calibration period (2002–08).

We calibrated the model to data between 2002, and 2008; model simulations were initiated in 1992 to allow for a 10-year burn-in period. We specified an initial population size of 32 889 (25 903 adults and 10 427 children aged 0–14 years), informed by local census data and projections of population growth. The values of many parameters in tuberculosis and HIV co-epidemics models are not known with certainty. Therefore, we adopted a Bayesian calibration approach^27^ to identify parameter sets that resulted in simulated trajectories with good fit to available epidemiological data (table 2). To implement this approach, we specified previous distributions for each parameter. Multiple parameters sets were randomly and independently selected from these distributions. We used each of these parameter sets to simulate epidemic trajectories, and measured the goodness-of-fit for each of these simulations against several calibration targets. These calibration targets were operationalised as the likelihood of recording the epidemiological data conditional on the simulated values. The appendix provides additional details about the likelihood function used and the methods to characterise the posterior parameter distributions. Figure 2 displays the fit of simulated trajectories against the calibration targets listed in table 2.

### Interventions

We used the model to project the effect of two targeted interventions: targeted active tuberculosis case finding and secondary isoniazid preventive therapy. For targeted active case finding we assumed that all adults who previously completed tuberculosis treatment were re-evaluated for active tuberculosis on average once per year and referred for tuberculosis treatment. We modelled targeted active case finding by increasing the rate of diagnosis, resulting in reductions in the average diagnostic delay, and the expected period of infectiousness (figure 1).

For secondary isoniazid preventive therapy, in the first year of intervention, we modelled a catch-up treatment campaign that reached 90% of individuals with previously completed tuberculosis treatment in the population. Subsequent to this catch-up period, we assumed that secondary isoniazid preventive therapy was offered to individuals after the completion of a full course of tuberculosis treatment and that an average of 90% of individuals completing treatment were enrolled. Secondary isoniazid preventive therapy reduces the rate of tuberculosis reactivation and the risk of progression to disease following re-infection. We allowed the preventive effects of secondary isoniazid preventive therapy to vary between 45% and 85%, a range informed by two previous studies.^30,31^ We assumed that the relative effect of secondary isoniazid preventive therapy was independent of HIV infection, but the absolute effect associated with this intervention remains greater for those with HIV in view of their higher reactivation rate and risk of progression.

Secondary isoniazid preventive therapy was intended as a lifelong intervention but we assumed that, on average, 15% of people currently on secondary isoniazid preventive therapy drop out every year (resulting in an expected duration of 6·6 years of secondary isoniazid preventive therapy), and that the protective effect of secondary isoniazid preventive therapy does not extend beyond the cessation of treatment.^32^

### Model outcomes and data analysis

We projected trends in tuberculosis incidence, prevalence, and mortality for 10 consecutive years—ie, 2016–25, under the baseline scenario and under two interventions scenarios: targeted active case finding alone and targeted active case finding plus secondary isoniazid preventive therapy. The effect of these interventions was defined as the cumulative number of tuberculosis cases and deaths averted during the 10-year period relative to the baseline scenario. The results are presented as the mean and 95% uncertainty intervals (the 2·5th and 97·5th percentiles of outcome values derived from 1000 simulated trajectories).

### Sensitivity and scenario analyses

To assess how sensitive the projected effect of targeted active case finding and secondary isoniazid preventive therapy was to input parameters of our model, we calculated partial rank correlation coefficients.^33,34^ The coefficients measure the correlation between an input parameter and the projected model outcome (number of incident tuberculosis cases averted) while adjusting for other parameters in the model. Additionally, we did the following types of scenario analyses: the projected effect of both targeted interventions under different periodicities of targeted active case finding (every 6 *vs* 12 and 24 months on average), different probabilities of secondary isoniazid preventive therapy enrolment (none *vs* 50%, 75%, and 90%), and different annual rates of drop-out from secondary isoniazid preventive therapy (5% *vs* 15% and 25%) were assessed. Furthermore, to provide additional insight on how well these targeted interventions might perform in communities with lower transmission rates, we report results for a hypothetical scenario where we reduced the force of infection by 50% relative to that in our study setting.

### Role of the funding source

The funders of the study had no role in study design, data collection, data analysis, data interpretation, or writing of the report. The corresponding author had full access to all of the data and the final responsibility to submit for publication.

## Results

We estimated that in 2016, 13% (95% uncertainty interval [UI] 11–16) of all adults in this population had previously been treated for active tuberculosis. The estimated prevalence of untreated tuberculosis was 2·2% (95% UI 0·9–3·8) in treatment-experienced adults, about 5·5 times higher than that in treatment-naive adults (0·4%, 0·1–0·8).

The identified parameter posterior distributions suggested that HIV uninfected treatment-experienced people were, on average, 1·6 times (95% UI 0·4–3·4) more susceptible to re-infection than were HIV uninfected people who were latently infected and tuberculosis treatment-naive. HIV uninfected adults who had completed tuberculosis treatment experienced, on average, a 35 times (95% UI 3·2–104·0) higher rate of tuberculosis reactivation than people who were latently infected and tuberculosis treatment-naive. The appendix provides posterior distributions of key parameters of the natural history of tuberculosis for treatment-experienced and treatment-naive individuals.

In the absence of targeted interventions, we projected 4457 (95% UI 2741–6723) incident tuberculosis cases and 623 (328–1031) tuberculosis-associated deaths between 2016 and 2025. In this period, 1423 (95% UI 670–22 311) incident tuberculosis cases will occur among adults who had completed a prior episode of treatment, representing 32% (20–39) of all incident cases.

**Figure 3:**
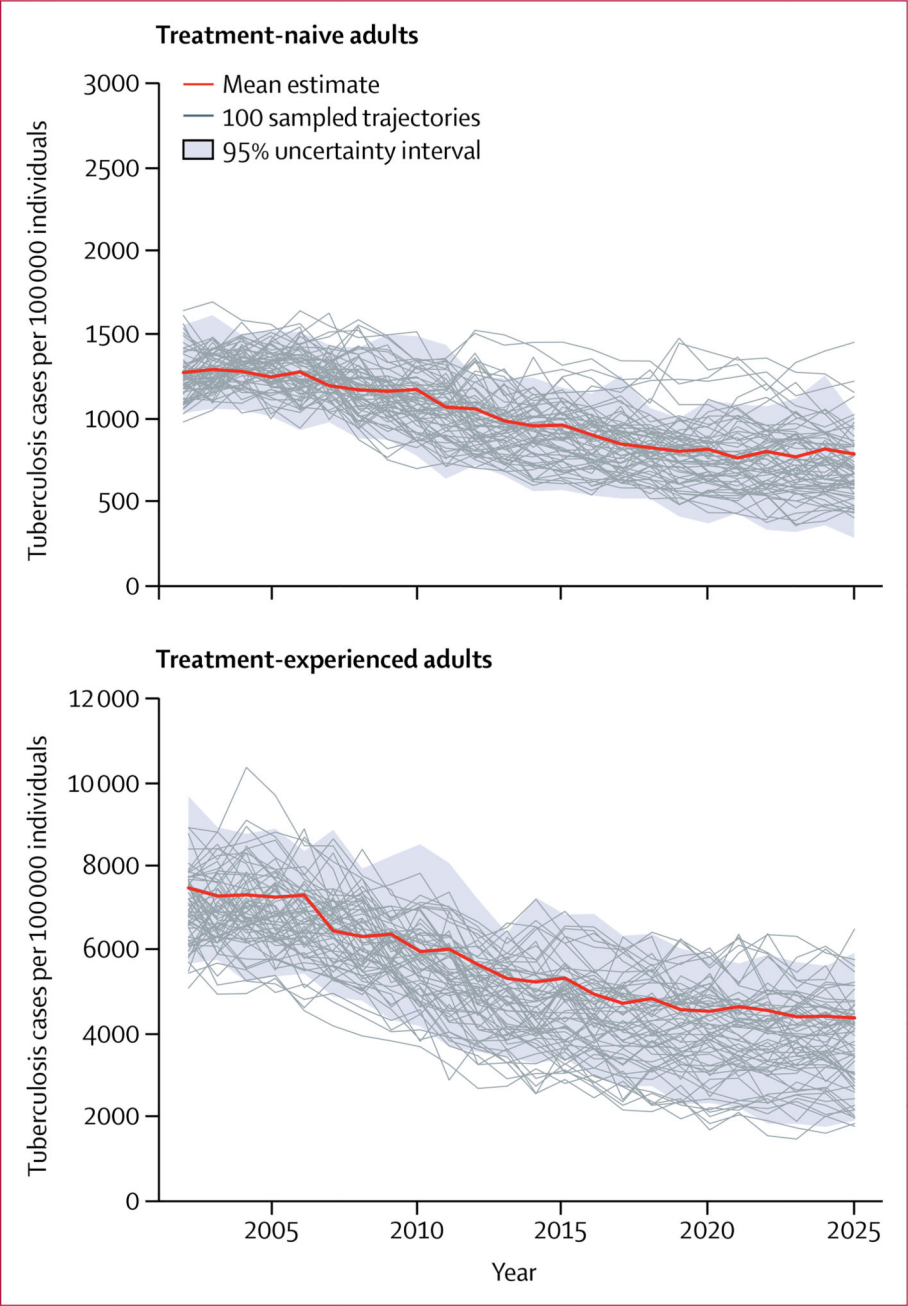
Tuberculosis incidence among treatment-naive and treatment-experienced adults between 2003 and 2025, projected under the baseline scenario Mean estimates (bold red line) represent the mean prediction at any given year. The 100 trajectories shown represent a random subset of the 1000 trajectories selected for analysis.

Figure 3 shows trends in tuberculosis incidence projected for treatment-naive and treatment-experienced adults over a 25-year period. Among treatment-naive adults, mean tuberculosis incidence per 100 000 people was 903 (95% UI 541–1147) in 2016 and was projected to decrease to 787 (287–1020) by 2025 (figure 3). Mean tuberculosis incidence among treatment-experienced adults was 4926 (95% UI 2949–6857) per 100 000 people in 2016, 5·5-times higher than among treatment-naive adults, and is expected to fall to 4353 (1874–5917) by 2025. The projected average annual decrease in tuberculosis incidence between 2016 and 2025 was 1·3% in treatment-naive and 1·2% in treatment-experienced adults.

With regards to the epidemiological effect of the interventions, our model suggests that annual targeted active case finding among individuals who had completed tuberculosis treatment would reduce the average duration of infectious disease in this group from 9·7 months (95% UI 2·3–17·5) to 5·0 months (1·9–7·1).

**Figure 4:**
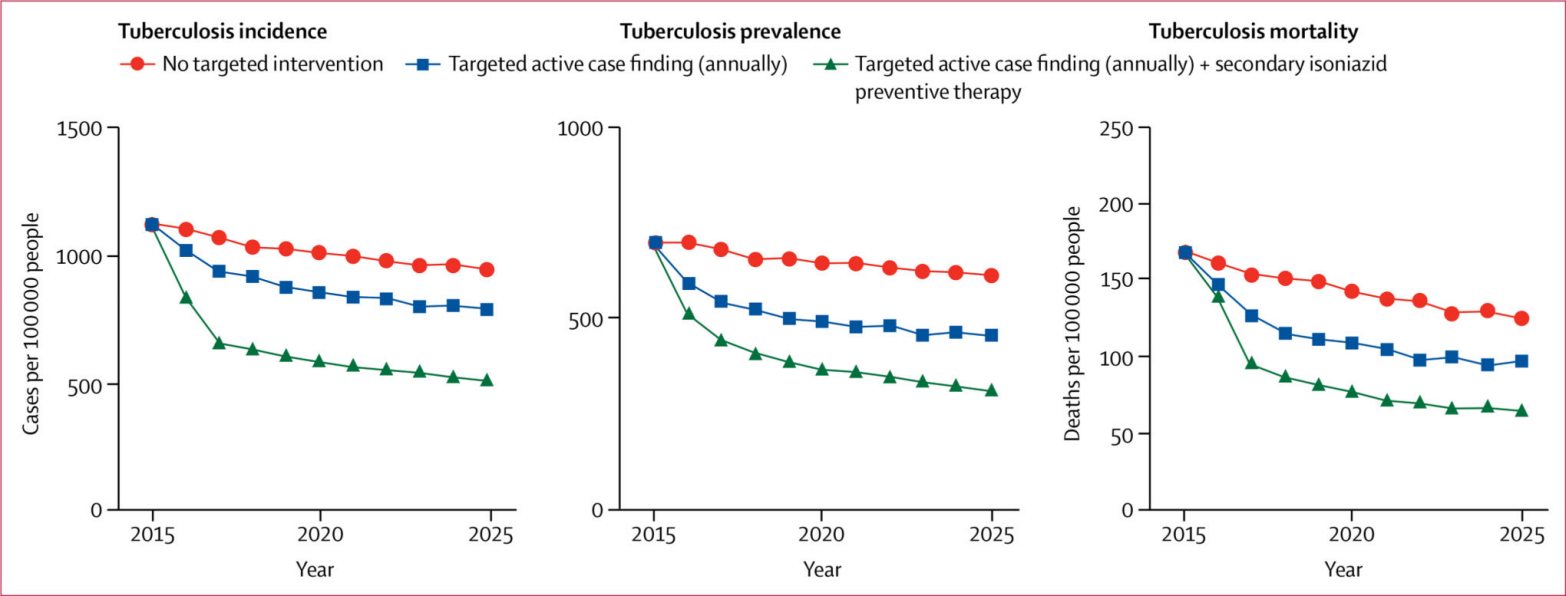
Projected epidemiological effect of interventions targeted to individuals with a history of previous complete tuberculosis treatment in a high-incidence setting in suburban Cape Town, 2016–2025

Figure 4 shows trends in tuberculosis incidence, prevalence, and mortality under the baseline scenario, under targeted active case finding alone, and under combined targeted active case finding and secondary isoniazid preventive therapy. The average annual decline in tuberculosis incidence between 2016 and 2025 relative to 2015 was 1·6% at baseline (no intervention), 3·0% under annual targeted active case finding, and 5·4% under annual targeted active case finding in combination with secondary isoniazid preventive therapy. Targeted active case finding alone would avert a total of 621 (95% UI 13–1355) incident tuberculosis cases between 2016 and 2025, 14% (0·4–28·0) of all incident tuberculosis cases projected under the baseline scenario. Over the same time period, targeted active case finding would avert a total of 138 (95% UI 13–296) tuberculosis deaths, 21% (2·5–39·0) of all tuberculosis deaths projected under the baseline scenario. The implementation of targeted active case finding in combination with secondary isoniazid preventive therapy would avert 1805 (95% UI 565–2952) incident tuberculosis cases between 2016 and 2025, 40% (21–56) of all incident tuberculosis cases projected under the baseline scenario. The combined targeted intervention would avert a total of 267 (95% UI 70–543) tuberculosis deaths, 41% (16–55) of all tuberculosis deaths projected under the baseline scenario.

Findings of sensitivity analysis showed that the projected effect of targeted active case finding and secondary isoniazid preventive therapy was most sensitive to the tuberculosis reactivation rate after completion of tuberculosis treatment, the time between tuberculosis disease onset and detection in the target group, the natural mortality rate in treatment-experienced relative to treatment-naive adults, and the efficacy of secondary isoniazid preventive therapy, among other parameters (appendix p 18). Lower periodicity of targeted active case finding (every 24 *vs* 12 months) and lower uptake of secondary isoniazid preventive therapy, as well as higher drop-out from secondary isoniazid preventive therapy, resulted in reduced effect (appendix p 19). In a hypothetical scenario in which we reduced the force of infection to 50% of the baseline value, we noted that annual targeted active case finding in combination with secondary isoniazid preventive therapy averted 34% (95% UI 16–54) of 2811 (1742–4503) incident tuberculosis cases and 36% (14–56) of 444 (231–760) tuberculosis deaths estimated at baseline (appendix p 19).

## Discussion

In this study, we used a calibrated population-based mathematical model to project the effect of two types of interventions targeted to previously treated people in a tuberculosis high-incidence setting. Our data suggest that, if targeted active case finding and secondary isoniazid preventive therapy were introduced to complement existing tuberculosis control efforts in this setting, the burden of tuberculosis could be substantially reduced. Our study supports the idea that efforts for prevention and prompt detection of recurrent tuberculosis^35^ could offer novel opportunities for tuberculosis control in settings of high tuberculosis incidence.

We propose these targeted control interventions during a time when untargeted efforts, such as population-wide enhanced case finding and household-based screening^8^ and mass isoniazid preventive therapy^9^ have yielded insufficient evidence of effect, and where novel approaches are urgently needed to reduce the burden of tuberculosis in communities most affected by the disease. Targeting control efforts to groups at high risk of tuberculosis could enable health services to make more efficient use of available resources. In many high tuberculosis prevalence settings, previously treated people can be easily identified and experience an elevated risk of tuberculosis,^16^ therefore they might be an attractive target for focused interventions.

We project that within 10 years in this setting, a combination of targeted active case finding and secondary isoniazid preventive therapy could avert more than a third of incident tuberculosis cases and tuberculosis deaths. Targeted active case finding alone could have a notable effect on tuberculosis prevalence and mortality, but is expected to have a smaller effect on incidence; our simulations suggest that a marked effect of targeted active case finding is achieved when it can be coupled with secondary isoniazid preventive therapy. Our projections show that much of the effect of targeted active case finding and secondary isoniazid preventive therapy accrues in the first few years after their implementation. The diminishing effect over time suggests a saturation effect, which might imply that such targeted interventions could be used within an adaptive control strategy.^21^

Our study constitutes a first step towards better understanding the effect of interventions targeted to previously treated people in high-incidence settings. However, several limitations must be noted. We applied our model to a specific setting with a high tuberculosis incidence and where high rates of recurrent tuberculosis due to relapse and re-infection had been previously reported.^12,14,36^ We note that the effect of interventions targeted at previously treated people, which we project for this setting, might not be easily generalised to other high-incidence settings for several reasons. High rates of recurrent tuberculosis have been reported from several other high-incidence settings.^10,11,13^ However, the population-level effect of targeted interventions will also depend on the size of the target group and their contribution to tuberculosis transmission in the population. In this particular setting, persistently high rates of incident tuberculosis have generated a large subgroup of people who had previously been treated for tuberculosis (about 10% of all adults) and who constitute a substantial proportion of the prevalent tuberculosis burden in the population (about 30% of prevalent cases).

Although our projections are consistent with the epidemiology of tuberculosis in other high-incidence communities in South Africa,^5,16^ we expect interventions among previously treated people to be less effective in settings with lower tuberculosis incidence, and where a smaller proportion of the tuberculosis burden is attributable to former tuberculosis patients. For example, previously treated people accounted for 4·1% of the adult population and for 13% of prevalent tuberculosis cases in Lusaka, Zambia,^6^ and for 1·5% and 15%, respectively, in Nigeria^37^—two settings with lower tuberculosis incidence than our study setting. Nonetheless, given that new approaches for tuberculosis control are most needed in areas where tuberculosis incidence has been persistently high, our results suggest that efforts to both prevent and rapidly detect and treat recurrent disease will produce important health benefits. In our scenario analysis, for which we lowered the force of infection by 50%, we noted that targeted active case finding in combination with secondary isoniazid preventive therapy reduced the expected number of incident tuberculosis cases and deaths to a lesser extent, but still averted a third of incident cases.

Differences in the prevalence of HIV in a population might influence the effect of interventions targeted to previously treated people in several ways. Communities with higher HIV prevalence might experience more recurrent tuberculosis given the elevated risk of re-infection with tuberculosis among HIV-infected individuals,^38^ and thus benefit more from similar interventions. Survival after a first tuberculosis episode might be reduced among those not on ART; those on ART may be subject to more regular clinical follow-up that would limit the benefit of additional case finding interventions in this group.

The population-level effect of targeted active case finding and secondary isoniazid preventive therapy will be dependent upon existing patterns of passive health-care seeking behaviour. In settings where there are longer delays to diagnosis, additional interventions to more rapidly identify and treat recurrent cases would be more effectual, whereas in areas where individuals self-present quickly after onset of symptoms, we would expect more modest returns from investment in combined targeted active case finding and secondary isoniazid preventive therapy interventions. This is consistent with our sensitivity analysis, which showed that the time to passive tuberculosis detection among treatment-experienced adults correlated with the projected effect.

Uncertainty around parameters of the natural history of tuberculosis, particularly those determining re-infection, disease progression, and mortality among previously treated individuals, leads to substantial uncertainty in the modelled outcomes. To avoid bias towards higher estimates of effect, we used conservative prior ranges of parameters for treatment-experienced adults, similar to those among treatment-naive adults. Specifically, we did not enforce higher susceptibility, lower partial immunity, or higher disease progression risk among those with a history of previous tuberculosis, but did allow posterior parameter values derived from calibration to vary by treatment history. While posterior distributions of our model are consistent with treatment-experienced people being more likely to become productively re-infected than treatment-naive people, we did not explicitly model differential risk of exposure, which could also be a mechanism driving increased risk of recurrent disease.^39^

Our study is further limited by uncertainty around the efficacy of secondary isoniazid preventive therapy towards preventing recurrent tuberculosis. As shown in our sensitivity analysis, higher effects of secondary isoniazid preventive therapy would result in higher effect at the population level. Only two studies—a randomised trial^30^ and a cohort study^31^—have assessed the effect of preventive therapy on recurrent tuberculosis. Both were limited in size and focused on people living with HIV. More available data from the field would improve our projections.

We used a simple mathematical model that does not enable us to explore specific intervention designs or consider many practical issues related to implementation. In particular, in our main analysis we assumed that interventions could be aggressively rolled out in these suburban settings—ie, that individuals with previous treatment could be effectively identified, enrolled, and screened for tuberculosis on average every 12 months, that 90% could be enrolled in secondary isoniazid preventive therapy upon completing treatment, and 15% would drop out from secondary isoniazid preventive therapy every year. Although we believe high coverage levels of the interventions could be achieved in this relatively small suburban setting, the effect of these interventions would clearly be lower if interventions were less vigorously applied or if some individuals were not reachable by the intervention.

In conclusion, our study provides impetus for further research to better understand the individual and population-level benefits of tuberculosis control interventions targeted at previously treated people. Studies and trials of the feasibility, safety, effect, and population-level effect of targeted active case finding and secondary isoniazid preventive therapy in previously treated people in high-incidence settings would be particularly useful. Other interventions to prevent recurrent tuberculosis such as adjuvant immunotherapy during tuberculosis treatment,^40^ extending the duration of tuberculosis treatment for certain high-risk patients,^34^ or post-treatment vaccination might be considered in the future. Further mathematical modelling, in which detailed costs of interventions are also included, would be useful for policy makers as they could establish whether such interventions are cost-effective and how investment in these approaches may compare with alternatives.

## Supplementary Material

Supplementary appendix

## Figures and Tables

**Table 1:** Selected model parameters describing differences between treatment-experienced and treatment-naive individuals

	Uniform prior distribution	Source
Relative infectiousness of treatment-experienced *vs* treatment-naive adults with active tuberculosis (ratio)	1·000 to 1·500	Assumption, based on findings from Marx et al^16^

Percentage reduction in susceptibility due to partial immunity (HIV-negative adults)		
During latent tuberculosis infection (tuberculosis treatment-naive)	0·370 to 0·870	Menzies et al,^20^ Dye and Williams,^22^ Dye and Espinal,^23^ Cohen et al,^24^ Dowdy and Chaisson^25^
After complete tuberculosis treatment	0·370 to 0·870	Assumption
After incomplete tuberculosis treatment	0·370 to 0·870	Assumption

Annual rate of tuberculosis reactivation (HIV-negative adults)		
Latent infection	0·0003 to 0·0024	Menzies et al,^20^ Dye and Williams,^22^ Dye and Espinal,^23^ Cohen et al,^24^ Dowdy and Chaisson^25^
Previously treated active tuberculosis	0·0003 to 0·048	Assumption

Baseline time (years) between onset of tuberculosis and detection (adults, independent of tuberculosis treatment history)		
Treatment-naive, HIV-negative adults and children	0·083 to 3·000	Menzies et al,^20^ Dye and Williams,^22^ Dye and Espinal,^23^ Cohen et al,^24^ Dowdy and Chaisson^25^
Treatment-experienced, HIV-negative adults	0·083 to 2·000	Assumption
HIV-positive adults	0·083 to 2·000	Assumption

Percentage tuberculosis treatment completion		
Treatment-naive adults	Time-varying	Estimated from tuberculosis treatment register database used in Marx et al.^14^
Adults after previous complete tuberculosis treatment	Time-varying	‥
Adults after previous incomplete tuberculosis treatment	Time-varying	‥
Probability of persistent active tuberculosis following incomplete tuberculosis treatment (adults, any HIV status)	0 to 0·200	Based on data from Marx et al^26^

**Table 2:** Overview of calibration targets and data sources

	Value	95% confidence interval	Source
Total population (2002)			
Adults	25 903	··	City of Cape Town*
Children	10 427	··	City of Cape Town

Percentage tuberculosis treatment-experienced adults (2002)	9·70	8·70–10·90	den Boon et al^28^

Percentage tuberculosis prevalence, treatment-naïve adults (2002)	0·51	0·26–0·76	den Boon et al^28^

Percentage tuberculosis prevalence, treatment-experienced adults (2002)	2·99	1·14–4·77	den Boon et al^28^

Percentage HIV prevalence, adults (2002)	5·20	··	Assumption, based on data from Western Cape Department of Health^29^

Number of children who started tuberculosis treatment (2002–08)	Time-varying	··	Tuberculosis treatment register database used in Marx et al.^14^

Number of treatment-naive adults who started tuberculosis treatment (2002–08)	Time-varying	··	Tuberculosis treatment register database used in Marx et al.^14^

Number of treatment-experienced adults who started tuberculosis treatment (2002–08)	Time-varying	··	Tuberculosis treatment register database used in Marx et al.^14^

* Unpublished end-of-year estimates (community level) from the 2001 South Africa population census provided by the City of Cape Town.

## Research in context

### Evidence before the study

Up to now, no empirical studies have been done of the population-level effect of interventions that aim to prevent recurrent disease or more rapidly detect tuberculosis in previously treated people. To establish whether population-based mathematical models have been employed to estimate the effect of tuberculosis interventions targeted to previously treated people, we did a PubMed search of relevant articles published in any language through March 7, 2017, using the search terms “(tuberculosis) AND (recurren* OR relapse OR reinfection OR re-infection OR re-treatment OR previous treatment) AND (model* OR simulation)”. We also reviewed titles and abstracts of mathematical modelling studies identified through an earlier comprehensive systematic literature review of studies describing mathematical and economic modelling of tuberculosis published through March 30, 2013 (conducted by Tuberculosis Modeling and Analysis Consortium [TB-MAC]). While mathematical models have considered the effect of improving treatment outcomes as a means of reducing relapse and associated transmission, none has addressed preferential targeting of tuberculosis control interventions to former tuberculosis patients.

### Added value of this study

We developed a transmission-dynamic mathematical model of the tuberculosis epidemic and calibrated it to epidemiological and demographic data from a setting with a high incidence of tuberculosis in suburban Cape Town, South Africa. High rates of recurrent tuberculosis and a high prevalence of tuberculosis in previously treated people have previously been reported from this setting. We presented estimates of the potential effect of tuberculosis interventions targeted to people who completed an episode of tuberculosis treatment and noted that targeted prevention and case finding efforts could yield substantial benefits for tuberculosis control at the population level.

### Implications of all the available evidence

Our results suggest substantial public health potential for control interventions targeted towards individuals with a history of previous tuberculosis treatment in settings with a high disease incidence. In these settings, previously treated people are especially attractive for targeted control interventions because they remain at an increased risk of active tuberculosis after apparent cure, contribute substantially to onward transmission, and should be readily identifiable by national tuberculosis programmes. Efforts to establish the feasibility and costs of such targeted interventions are needed to establish their cost-effectiveness in tuberculosis and HIV endemic settings.
