# Supplementary material for: Tuberculosis control interventions targeted to previously treated people in a high-incidence setting: a modelling study
Source: Lancet Glob Health. Author manuscript; Available in PMC 2018 Apr 1. (PMC5849574; doi:10.1016/S2214-109X(18)30022-6)
Supplement: Supplementary appendix [file NIHMS946270-supplement-Supplementary_appendix.pdf]

THE LANCET
Global Health

Supplementary appendix

This appendix formed part of the original submission and has been peer reviewed. We post it as supplied by the authors.

Supplement to: Marx FM, Yaesoubi R, Menzies NA, et al. Tuberculosis control interventions targeted to previously treated people in a high-incidence setting: a modelling study. *Lancet Glob Health* 2018; published online Feb 19. http://dx.doi.org/10.1016/S2214-109X(18)30022-6.

APPENDIX

Tuberculosis control interventions targeted to previously treated people in a high-incidence setting: a modelling study

Florian M. Marx, Reza Yaesoubi, Nicolas A. Menzies, Joshua A. Salomon, Alyssa Bilinski, Nulda Beyers, Ted Cohen

Contents

S1. Study setting ... 2

S2. Model structure ... 2

S3. Model parameterization ... 3

S3.1. Demographics ... 3

S3.2. Natural history of TB ... 4

S3.3. Natural history of TB: Characteristics of treatment-experienced adults ... 5

S3.4. TB case detection and treatment ... 6

S3.5. TB-associated (excess) mortality ... 7

S3.6. Natural history of HIV infection ... 8

S3.7. Initiation of antiretroviral treatment among HIV-infected adults ... 9

S4. Simulation approach ... 9

S5. Model calibration ... 11

S5.1. Calibration data sources ... 11

S5.2. Calibration procedure ... 13

S6. Outcome definitions and data analysis ... 13

S7. Posterior estimates for the natural history of TB by history of TB treatment ... 14

S8. Sensitivity and scenario analyses ... 14

Appendix References ... 20

S1. Study setting

Our study focuses on two adjacent suburban communities with a high tuberculosis (TB) burden in Cape Town, South Africa, covering an area of 3.4 km², and with a total population of 39,930 people in 2011. The internationally-endorsed TB control strategy (DOTS) was introduced in these communities in 1996. In the first year of the program, the rate of notified TB (all forms) was 1,340 cases per 100,000 residents.[1] Treatment success rates were initially low but increased rapidly and exceeded 80% amongst smear-positive TB cases in 2003.[2] However, persistently high annual rates of infection (estimated 3·7% in 1999 and 4·1% in 2005[2]) suggest that control measures, while improving individual outcomes, did not reduce transmission.[3] High local rates of recurrent TB after previous successful treatment[4-6] and after loss to follow-up from treatment[7] have also been reported; a lung health survey conducted in 2001 identified a high prevalence of undetected TB among previously treated residents.[8]

S2. Model structure

Childhood subcomponent: At birth, individuals enter the childhood subcomponent of the TB model (Figure S1) in the susceptible state, where they face a time-varying risk of infection, conditional on the force of infection which is dependent on the total number of infectious cases (adults and children) at time t. Upon primary infection, children either progress rapidly to infectious TB or reach a latently infected (non-infectious) state. Children may remain in the latent state, or their infection may reactivate and progress to infectious TB. They may also become reinfected and either rapidly progress to infectious disease or remain in the latent state. Upon infectious disease, children may move into a recovered state after being found and treated.
At any state, children may leave the model subcomponent into the main (adult) component at rates reflecting their age progression beyond 14 years (Figure S1). Specifically, children transit from the susceptible state into the adult treatment-naïve susceptible state, from the latently infected state into the adult treatment-naïve latently infected state, and from the infectious state into the adult treatment-naïve infectious state. We assume that treatment of childhood TB is always complete, thus, children in the recovered state move into the adult latently infected after complete treatment state.

Main component (adults): Treatment-naïve susceptible adults transition from the susceptible state to the latently infected state or directly into the infectious TB state after primary infection (Figure 1, main manuscript). Latently infected treatment-naïve adults may experience reactivation disease and transition into the infectious TB state. If reinfected while in the latently infected state, they may progress to infectious disease or remain latently infected. Treatment-naïve infectious adults may be identified and move into either of the two treatment compartments (treatment that is completed, treatment that is incomplete). The transition into these two treatment states is determined by the case finding rate and the proportion of complete treatment among new (i.e. previously treatment-naïve) TB cases estimated for the study setting. Individuals in the incomplete treatment state move into a treatment-experienced latently infected state or, upon continuous infectious TB, directly into the infectious TB state. From latent infection, they may progress to infectious TB either via disease reactivation or following reinfection. Upon complete treatment,

all adults transition to a latently infected state (i.e. consistent with many TB models, we assume that sterilizing cure is not achieved). We introduced two different states of latent infection for those individuals completing treatment. This allows us to distinguish whether individuals were enrolled in 2°IPT. Latently infected adults after complete treatment may progress to infectious disease either via reactivation or following reinfection. Similar to treatment-naïve infectious cases, cases occurring after either incomplete or complete treatment move back into the two treatment states at rates determined by case finding rates and the proportion of complete treatment estimated for the study setting. We implemented an active case finding rate, incremental to the passive case detection rate, to simulate TACF among adults who previously completed TB treatment. Individuals may exit the model due to death from any state, with additional excess mortality rates due to TB disease and HIV infection implemented in out model.

Model subdivisions for HIV co-infection and antiretroviral treatment: Upon HIV infection (Figure 1, main manuscript), HIV-negative adults transit into a non-immunocompromised HIV infected state, and upon progression, into an immunocompromised subdivision. Upon initiation of antiretroviral treatment (ART), individuals in either of the two prior HIV-positive subdivisions may transit into a fourth subdivision. Once initiated on ART, individuals were assumed to stay on ART. We did not model HIV in children.

S3. Model parameterization

Parameter values and ranges used in the model along with their sources are provided in the subsequent sections and Tables S1-S14. Rates shown are per year unless otherwise specified.

S3.1. Demographics

Estimates for demographic parameters are based on data from the Tygerberg sub-district of Cape Town in which the study setting is situated. We assumed a constant birth rate throughout the study period which was estimated by dividing the number of life births in the study setting reported for the year 2003[9] by the projected population in 2003 (Table S1). Estimates of the natural death rates among children 0-14 years of age were derived from unpublished mortality data (for 2011) provided by the City of Cape Town Directorate of Health (Table S1). In the absence of published data, we derived an estimate of the natural mortality rate among adults through calibration, allowing for a 1·0% annual population growth, consistent with unpublished census data for the study setting (Table S1). We assumed that the rate of natural death among treatment-experienced adults was between equal and 5-times higher compared to treatment-naïve adults. This range takes into account the possibility that mortality among former TB patients may be higher[10-12] due to a variety of factors such as lung impairment and chronic pulmonary disease[13] and an elevated risk of death from lung cancer[14] compared to individuals without a history of TB.
We assumed that on average, a child would be in contact with 40 other children and 9 adults per day, and an adult would be in contact with 15 adults and 9 children per day.[15]

Table S1: Model Parameters – Demographics

Measure	Value [Interval]	Source
Annual per capita birth rate	0·0229	[9]
Annual population growth	1·0%	estimated from unpublished census data, City of Cape Town
Annual natural death rate among children (<15 years)	0·0017	estimated from unpublished census data, City of Cape Town
Annual natural death rate among adults (≥15 years)	[0·0086-0·0096]	Experiments with the model
Natural death rate ratio, TB treatment-experienced adults to treatment-naïve adults	[1-6]	assumption

S3.2. Natural history of TB

Estimates for transition rates between TB-related states were derived from the published literature, where available (Tables S2-S5). In accordance with prior modeling studies, we considered that distant prior (latent) infection would lead to partial immunity reducing the risk of becoming reinfected (Table S4). Parameters for HIV-infected adults take into account that HIV alters the natural history of TB. Specifically, HIV-infected individuals are subject to a higher probability of fast progression to active TB following infection[16,17] (Table S2) and a higher probability of reactivation of latent infection[18] (Table S3).

We assumed that children were less likely to transmit TB by the ratio 0·12 [0·034-0·305] (compared to treatment-naïve adults) that was based on the probability of smear-positive TB among children and adults estimated in a recent meta-analysis.[19]

Table S2: Model Parameters - Probability of Fast Progression to Active TB Upon Primary Infection

Subgroup	Value [Interval]	Source
Adults, susceptible/treatment-naïve/HIV-	0·115 [0·09-0·14]	[20-22]
Adults, susceptible/treatment-naïve/HIV+/non-immunocompromised	0·33 [0·18-0·51]	[20-22]
Adults, susceptible/treatment-naïve/HIV+/ immunocompromised	0·805 [0·75-0·91]	[20-22]
Children, susceptible	0·118 [0·09-0·14]	estimated from [23]

Table S3: Model Parameters - Rate of Reactivation of latent TB infection

Subgroup	Value [Interval]	Source
Adults, latently infected/treatment-naïve/HIV-	0·001 [0·0003-0·0024]	[21,22,24,25]
Adults, latently infected/treatment-naïve/HIV+/non-immunocompromised	0·003 [0·001-0·006]	[21,22,24,25]
Adults, latently infected/treatment-naïve/HIV+/ immunocompromised	0·1275 [0·080-0·200]	[21,22,24,25]
Children, latently infected	0·001 [0·0003-0·0024]	assumption

Table S4: Model Parameters – Percent Reduction in Susceptibility due to Partial Immunity afforded by Prior Infection (treatment-naïve)

Subgroup	Value [Interval]	Source
Adults, latently infected/HIV-	0·65 [0·37-0·87]	[22,24,26-28]
Adults, latently infected/HIV+/non-immunocompromised	0·45 [0·23-0·68]	[22,24,26-28]
Adults, latently infected/HIV+/ immunocompromised	0·25 [0·14-0·39]	[22,24,26-28]
Children, latently infected	0·65 [0·37-0·87]	assumption

Table S5: Model Parameters – Rate of Natural Recovery among Undetected Active TB Cases

Subgroup	Value [Interval]	Source
Adults, infectious/treatment-naïve/HIV-	0·2 [0·15-0·25]	[21,22,26,29]
Adults, infectious/treatment-naïve/HIV+/non-immunocompromised	0·1 [0·06-0·16]	[21,22,26,29]
Adults, infectious/treatment-naïve/HIV+/ immunocompromised	0	[21,22,26,29]
Children, infectious	0·2 [0·15-0·25]	assumption

S3.3. Natural history of TB: Characteristics of treatment-experienced adults

The model allows for specific characteristics in the natural history of TB among individuals previously treated for the disease. In the absence of published estimates for many of these parameters, we specified prior parameter ranges and derived posterior parameter values through calibration (see below).

We assumed that TB treatment-experienced people were equally likely to be exposed to an individual with infectious TB in the community compared with treatment-naïve people. However, we allowed treatment-experienced adults to differ from treatment-naïve, latently infected adults in terms of their risk of becoming reinfected upon exposure. This was achieved through differential parameters for partial immunity towards reinfection among treatment-experienced and treatment-naïve people derived through calibration (same prior ranges; Table S6, see Table S4 for comparison). Rates of reactivation TB after complete and incomplete treatment were derived from calibration. To account for the possibility of higher reactivation rates after prior treatment for active TB, we specified prior parameter ranges for reactivation rates (Table S7) with the lower boundary being equal and the upper boundary 20-times higher than that for reactivation of distant prior latent infection (compare Table S3).

Based on findings from prevalence surveys that treatment-experienced cases of TB were more likely to be coughing and to be smear-positive[30], we assumed that treatment-experienced TB cases were equal to 1·5-times more likely to transmit TB compared to treatment-naïve TB cases in terms of their potential to transmit TB.

Individuals with incomplete treatment may continue to suffer from infectious disease. Based on data from a retrospective cohort study conducted previously in the study setting[7], we assumed that between 0 and 20% of those who were lost to follow-up during treatment remained infectious and thus moved directly into the compartment of infectious TB (Table S8). We assumed that recurrent cases of TB after previous complete or incomplete treatment were equally likely to transmit compared with cases of a first episode of TB.

Table S6: Model Parameters –Percent Reduction in Susceptibility due to Partial immunity after (previously treated) active TB

Subgroup	Value [Interval]	Source
Adults, latently infected/prior complete or incomplete treatment/HIV-	- [0·37-0·87]	22,24,26-28
Adults, latently infected/ prior complete or incomplete treatment/HIV+/non-immunocompromised	- [0·23-0·68]	22,24,26-28
Adults, latently infected/ prior complete or incomplete treatment/HIV+/ immunocompromised	- [0·14-0·39]	22,24,26-28

Table S7: Model Parameters – Rate of Reactivation of active TB after treatment

Subgroup	Value [Interval]	Source
Adults, prior complete treatment/HIV-	0·001 [0·0003-0·048]	see: S3.2
Adults, prior incomplete treatment/HIV-	0·001 [0·0003-0·048]	see: S3.2
Adults, prior complete treatment /HIV+/ non-immunocompromised	0·003 [0·001-0·12]	see: S3.2
Adults, prior incomplete treatment /HIV+/non-immunocompromised	0·003 [0·001-0·12]	see: S3.2
Adults, prior complete treatment/HIV+/ immunocompromised	0·1275 [0·080-4·00]	see: S3.2
Adults, prior incomplete treatment /HIV+/ immunocompromised	0·1275 [0·080-4·00]	see: S3.2

Table S8: Model Parameters – Probability of Persistent Active TB Following Incomplete Treatment

Subgroup	Value [Interval]	Source
Adults, prior incomplete treatment/any HIV-status	[0-0·20]	based on data from [7]

S3.4. TB case detection and treatment

Parameters for TB case detection rates were derived from calibration. We allowed for shorter times to detection assuming that people who had experienced TB treatment may seek care more promptly than those without previous TB treatment. We also assumed shorter times to detection for HIV-infected people (Table S9). The prior ranges used were informed by estimates of infectious disease duration before detection from previous studies in South Africa[31] and Zimbabwe[32].

We assumed that TB cases on treatment are non-infectious, i.e. they do not contribute to transmission. The duration of complete treatment among new and re-treatment cases was estimated from treatment register data (Table S10). We assumed that treatment is either complete or incomplete. Proportions of complete treatment among treatment-naïve and treatment-experienced people between 1996 and 2008 were estimated from the TB register database (Table S11). For the years following 2008, we randomly sampled treatment completion probabilities from a uniformly distributed range of probabilities specified by the 1996 to 2008 data.

Table S9: Model Parameters – Baseline time between disease onset and detection (years)

Subgroup	Value [Interval]	Source
Adults, infectious/treatment-naïve/HIV-	[0.083-3]	assumption
Adults, infectious/ or prior complete or incomplete treatment/HIV-	[0.083-2]	assumption
Adults, infectious/prior treatment-naïve or prior complete or incomplete treatment/HIV+	[0.083-2]	assumption
Children, infectious	[0.083-3]	assumption

Table S10: Model Parameters – Duration of treatment (years)

Subgroup	Value [Interval]	Source
Adults, complete treatment	0·50 (0·47-0·57)	TB program data
Adults, incomplete treatment	0·42 (0·31-0·52)	TB program data

Table S11: Probability of complete treatment

Subgroup	Year							Source
	2002	2003	2004	2005	2006	2007	2008	
Adults, treatment-naïve	91 (87-94)	98 (95-99)	97 (94-98)	94 (90-96)	97 (94-98)	99 (96-99)	98 (96-99)	TB program data
Adults, prior complete treatment	92 (82-97)	92 (83-96)	92 (85-96)	94 (86-97)	88 (79-94)	94 (87-98)	89 (80-94)	TB program data
Adults, prior incomplete treatment	60 (37-79)	84 (60-95)	82 (56-94)	65 (40-84)	83 (58-95)	55 (33-75)	77 (46-93)	TB program data

S3.5. TB-associated (excess) mortality

We considered excess mortality rates (incremental to natural death rates) for two different groups, those with untreated active (infectious) TB (Table S12) and those on TB treatment (Table S13). We assumed that the excess mortality rate among HIV-infected non-immunocompromised adults and those HIV-infected on ART was similar to that among HIV-uninfected individuals. We further assumed that the excess mortality rate among untreated children was similar to that among HIV uninfected adults, and that children would not die from TB while on treatment (Table S13).

Table S12: Model Parameters – Rate of TB-associated (excess) mortality rate, untreated TB

Subgroup	Value [Interval]	Source
Adults, infectious/prior treatment-naïve or prior complete or incomplete treatment/HIV-	0·28 [0·20-0·37]	[21,22]
Adults, infectious/prior treatment-naïve or prior complete or incomplete treatment/HIV+/non-immunocompromised	0·28 [0·20-0·37]	assumption, see S3.5
Adults, infectious/prior treatment-naïve or prior complete or incomplete treatment/HIV+/ immunocompromised	0·80 [0·47-1·27]	[22,33,34]
Adults, infectious/prior treatment-naïve or prior complete or incomplete treatment/HIV+/ART	0·28 [0·20-0·37]	assumption, see S3.5
Children, infectious	0·28 [0·20-0·37]	assumption, see S3.5

Table S13: Model Parameters – Rate of TB-associated (excess) mortality rate, on TB treatment

Subgroup	Value [Interval]	Source
Adults, infectious (any subcategory)	0·056 [0·047-0·070]	estimated from TB program data
Children, infectious	0	assumption

S3.6. Natural history of HIV infection

Adults may be infected with HIV at any state in the model and move across the HIV subdivisions. The rate of HIV transmission in the adult population was derived from calibration. Rates of progression from non-immunocompromised to immunocompromised HIV and that of HIV-associated excess mortality among non-immunocompromised people were estimated from data published in the literature (Table S14). The distinction between *non-immunocompromised* and i*mmunocompromised* HIV-infected adults was made on the basis of CD4 count cut-off level of <350/mm³. HIV-associated excess mortality among immunocompromised people was calculated from estimates of survival time among HIV-infected people not on ART, assuming that 75% of these died from HIV-related causes other than TB. It was assumed that all children in the study setting were and remained HIV uninfected.

Table S14: Model Parameters – HIV-progression, HIV-associated mortality and effect of ART

Measure	Value [Interval]	Source
Annual rate of progression to immunocompromised HIV from non-immunocompromised HIV	0·142 [0·135-0·149]	35
Survival time of HIV-infected people not on ART (years)	10·2 [9·7-10·5]	36
Annual non-immunocompromised HIV-associated excess mortality rate	0·008 [0·005-0·012]	22,37-41
Annual immunocompromised HIV-associated excess mortality rate	0·068 [0·062-0·074]	calculated from estimated survival time, see above
Annual HIV-associated excess mortality rate while on ART	0·008 [0·005-0·012]	22,37-41
Effectiveness of ART in reversing effect of HIV on TB natural history (compared to the HIV+/non-immunocompromised state, excluding mortality)	0·69 [0·47-0·81]	42

S3.7. Initiation of antiretroviral treatment among HIV-infected adults

Assumptions were made to consider ART initiation among HIV-infected people in the study setting.

ART among immunocompromised adults not on TB treatment. We assumed a (historical) rate of ART initiation among immunocompromised people of 0·1 per year in 2004, the year of ART roll-out in Cape Town, and a linear increase of this rate to 0·3 per year in 2016, after which the rate remains constant.

ART among non-immunocompromised adults not on TB treatment. Considering the possibility that ART is also offered to HIV-infected people above a CD4 count of 350mm^3, we assumed a rate of ART initiation among non-immunocompromised people of 0·02 per year in 2004, and a linear increase of this rate in the following years to 0·1 per year in 2016, after which the rate remains constant.

ART among immunocompromised and non-immunocompromised adults starting TB treatment. In line with national TB guidelines for South Africa[43], it was considered that ART is also initiated when HIV-infected people start TB treatment. We assumed that ART was initiated among 10% of HIV-infected individuals starting TB treatment. This proportions increases linearly to 30% until 2016 and remains constant at 30% in the following years. We assumed that ART was initiated at the start of TB treatment but was not initiated at a later stage during the course of TB treatment.

Figure S2 shows the projected coverage of ART among treatment-naïve and treatment-experienced HIV-infected adults (not on TB treatment) over time derived from our model.

S4. Simulation approach

Let $\lambda_{i \leftarrow i'}$ denote the rates at which members of age group $i \in \{\text{Ch}, \text{Ad}\}$ contact members of age group $i' \in \{\text{Ch}, \text{Ad}\}$ and let $H = \{I_{TN}, I_{TI}, I_{TC}\}$ denote the set of adult compartments with infectious status (TN = treatment-naïve, TI = prior incomplete treatment and TC = prior complete treatment). We used $N_{\text{Ch}}(t)$ and $N_{\text{Ad}}(t)$ for the number of children and adults at time t, and $N_h(t)$ for the number of population members in model compartment h.

We defined the force of infection for susceptible and latent children ($h \in$ {S_{Ch}, L_{Ch}}) at time t as:

$$F_h(t) = \beta_h \left(\lambda_{Ch \leftarrow Ch} \frac{N_{I|Ch}(t)}{N_{Ch}(t)} + \sum_{h' \in H} \lambda_{Ch \leftarrow Ad} \frac{N_{h'}(t)}{N_{Ad}(t)}\right), \quad (1)$$

and for susceptible and latent adults ($h \in$ {S_{TN}, L_{TN}, L_{TC}, L_{TI}}) as:

$$F_h(t) = \beta_h \left(\lambda_{Ad \leftarrow Ch} \frac{N_{I|Ch}(t)}{N_{Ch}(t)} + \sum_{h' \in H} \lambda_{Ad \leftarrow Ad} \frac{N_{h'}(t)}{N_{Ad}(t)}\right). \quad (2)$$

In above equations, β_h is the transmission parameter in compartments $h \in$ {S_{Ch}, L_{Ch}, S_{TN}, L_{TN}, L_{TC}, L_{TI}}, where S denotes susceptible, and L denotes latently infected. Based on existing survey data,[15] we assumed $\lambda_{Ch \leftarrow Ch} = 4.7$, $\lambda_{Ch \leftarrow Ad} = \lambda_{Ad \leftarrow Ch} = 3.1$ and $\lambda_{Ad \leftarrow Ad} = 10.7$.
To generate epidemic trajectories for this model, we use Monte Carlo simulation. Consider a particular compartment Z in which members may depart due to J events. For example, members of L_{TN} compartment may leave due to reactivation of latent infection, reinfection, or natural death (i.e. $J = 4$) (see Figure 1). If the number of individuals in compartment Z at time t is $Z(t)$, then the number of individuals that leave this compartment due to events $j \in \{1,2, \dots, J\}$ follows a multinomial distribution with total counts of $Z(t)$ and probabilities $(p_0, p_1, p_2, \dots p_J)$, where $p_0 = 1 - e^{\sum_{j=1}^{J} \mu_j \Delta t}$ is the probability of not leaving the compartment Z during $[t, t + \Delta t]$, and $p_j = \frac{\mu_j}{\sum_{j=1}^{J} \mu_j \Delta t} e^{\sum_{j=1}^{J} \mu_j \Delta t}$ is the probability of leaving the compartment Z during $[t, t + \Delta t]$ due to event $j \in \{1,2, \dots, J\}$. Having obtained the realizations for the number of individuals who move from one compartment to another during $[t, t + \Delta t]$, we can then update the number of individuals in each compartment at time $t + \Delta t$.

Model Initialization

In the absence of published estimates for the prevalence of HIV, active TB and treatment-experienced individuals in the year 1992 (which marks the start of our simulation warm-up period), we determined the initial size of model compartments based on the following:

1. Prevalence of immunocompromised and non-immunocompromised HIV is sampled, respectively, from uniform distributions U [%3·5; %5·0] and U [%0·5; %1·0]. The prevalence of HIV-negative was set to 1 minus the sum of the above two samples.
2. Prevalence of the treatment-experienced within each HIV subgroup was sampled from the uniform distribution U [%6·0; %10·0]. The proportion of treatment-experienced with history of complete or incomplete TB treatment was set to be equal.
3. Within the HIV-negative subgroup:
    a. the prevalence of active TB was sampled from U [%0·4; %0·6] for treatment-naïve subgroup, and from U [%1·0; %10] for treatment-experienced subgroup;
    b. the prevalence of latent-TB among treatment-naïve was sampled from U [%40; %60].
4. Within non-immunocompromised HIV+ subgroup,
    a. the prevalence of active TB was sampled from U [%0·5; %2·0] for treatment-naïve subgroup and from U [%1·0; %10] for treatment-experienced subgroup;

b. the prevalence of latent-TB among treatment-naïve was sampled from U [%55; %65]

5. Within immunocompromised HIV+ subgroup,
   a. the prevalence of active TB was sampled from U [%0·5; %2] for treatment-naïve subgroup and from U [%1·0; %10] for treatment-experienced subgroup;
   b. the prevalence of latent-TB among treatment-naïve was sampled from U [%55; %65]
6. Among children:
   a. Prevalence of active TB was sampled from U [%0·1; %1·0],
   b. Prevalence of latent-TB was sampled from U [%30; %70],
   c. Proportion recovered was sampled from U [%2·0; %10],
   d. Proportion susceptible was set to 1 minus the sum of the three samples above.

The initial size of compartments representing "on TB treatment" was assumed to be zero at the beginning of the simulation period.

S5. Model calibration

S5.1. Calibration data sources

We calibrated the model to data from three main sources. Population census data provided by the City of Cape Town were used to obtain estimates of the size and age structure (i.e. children vs. adults) of the population in the study setting. Data from a lung health prevalence survey conducted in the study setting in 2002[8] were used to derive estimates of the proportion of adults with a history of previous TB treatment and of the prevalence of TB among treatment-naïve and treatment-experienced adults in 2002. Estimates of the crude prevalence of TB by treatment history were calculated from[8] by dividing each, the number of treatment-naïve and treatment-experienced adults detected with culture-confirmed TB by the total number of adults in the survey sample multiplied by each, the proportion of treatment-naïve and treatment-experienced adults in the survey sample, respectively. Finally, we accessed TB treatment data from an electronic TB treatment register database that had been cleaned for duplicate entries and assessed for data consistency to obtain the number of new and previously treated TB cases registered for treatment in the study setting. The proportion of new and previously treated TB patients with complete TB treatment was estimated among new and previously treated TB cases by dividing the number of TB cases with documented treatment outcome success by the total number of patients with either treatment success or treatment default (loss to follow-up; defined by treatment interruption for at least two consecutive months) in that particular year (i.e. thereby excluding TB cases with treatment failure, transfer out or unknown treatment outcome from the denominator).

To estimate parameters of HIV transmission in the community, we calibrated the model to an estimated HIV prevalence of 5·2% (4·0%-6·0%) among adults living in the study setting in 2002, assuming that HIV-prevalence was half of the 2002 antenatal survey estimate for the greater Tygerberg East Sub-district.[44]

Calibration targets, data sources, and specified feasible ranges are shown in Tables S15-S17.

Table S15: Calibration Targets for 2002

Target	Value [Interval]	Source
Number of adults in the study setting	25,903	City of Cape Town*
Number of children in the study setting	10,427	City of Cape Town*
Percentage treatment-experienced, all adults	9·7 [8·7-10·9]	8
Percentage prevalent TB, treatment-naive adults	0·51 [0·26-0·76]	8
Percentage prevalent TB, treatment-experienced adults	2·99 [1·14-4·77]	8

* Unpublished end-of-year estimates (community level) from the 2001 South Africa population census provided by the City of Cape Town.

Table S16: Time-varying calibration targets (2002 -2008)

Target	Value [Interval]							Source
	2002	2003	2004	2005	2006	2007	2008	
Number of treatment-naïve adults starting TB treatment	172	234	200	224	216	233	210	TB treatment register database [6]
Number of treatment-experienced adults starting TB treatment	105	119	130	109	130	126	137	TB treatment register database [6]
Number of notified TB cases, children	82	60	66	69	73	77	69	TB treatment register database [6]
Percentage HIV-positive, all adults	5·2 [4-6]	- [4-6]	- [4-6]	- [4-6]	- [4-6]	- [4-6]	- [4-6]	estimated from [44]

Table S17: Specified feasible ranges for calibration targets

Target	Feasible Range
Number of adults in the study setting	24,000 - 30,000
Number of children in the study setting	10,000 - 12,500
Percentage treatment-experienced, all adults	5 - 15
Percentage prevalent TB, treatment-naive adults	0 - 1·0
Percentage prevalent TB, treatment-experienced adults	0 - 6·0
Percentage HIV-positive, all adults	2·6 - 10·4

S5.2. Calibration procedure

The goal of model calibration is to use the observations gathered throughout the epidemic to reduce the uncertainty around model input parameters. We used a Bayesian calibration approach[45] where the likelihood of observations in Tables S15-16 are measured using the probability distributions described below. For a given simulated trajectory:

1. The likelihood of the observed adult population size in each year (Table S15) is measured by a normal distribution with mean equal to the adult population size generated by the simulated trajectory. In the absence of sampling distribution for the estimated population size, we approximated the standard division of these normal distributions by $0.05N_t/z_{1-\alpha/2}$ where N_t is the adult population size in year t and $z_{1-\alpha/2}$ is the $(1-\alpha/2)$ upper critical value of a standard normal distribution. We chose $\alpha = 0.05$ ($z_{1-0.05/2}$=1.96). The likelihood of observed population of children is measured using the same approach.
2. The likelihood of observed prevalence of treatment-experienced adults is measured by a binomial distribution where the number of trials is set to the number of population-based survey participants and the probability of success is set to the prevalence of treatment-experienced adults projected by the simulated trajectory. We approximate the number of survey participants from the reported confidence intervals $[L, U]$ (see Table S15) by solving $\frac{U-L}{2} = z_{1-\alpha/2}\sqrt{\frac{1}{n}\hat{p}(1-\hat{p})}$ for n, where $\hat{p}$ is the estimated prevalence provided in Table S15. The likelihood of observed HIV prevalence, percentage prevalent TB among treatment-naive adults and percentage prevalent TB among treatment-experienced adults are calculated using the approach described above.
3. The likelihood of the observed number of treatment-naïve adults starting TB treatment in each year (Table S16) is measured by a binomial distribution where the number of trials is set to the population size of treatment-naïve adults produced by the simulated trajectory and the probability of success is set to proportion of treatment-naïve adults who started TB treatment in that year of the simulation. The likelihoods of the observed number of treatment-experienced adults starting TB treatment and the number of notified cases of pediatric TB are calculated in the same way.

S6. Outcome definitions and data analysis

We projected trajectories of TB incidence, prevalence and mortality. Incident TB was defined in our model as the number of adults and children, regardless of treatment history and HIV status, who transitioned into any of the infectious TB compartments; individuals remaining infectious after incomplete treatment were not counted in incidence estimates. Prevalent TB was defined as the number of adults and children in any of the infectious compartments at a particular point in time. TB mortality was defined as the number of adults and children who died while either in any of the infectious or TB treatment compartments.

Best estimates of incidence, prevalence and mortality were derived by calculating the mean of values projected from the 1,000 sampled model trajectories. We calculated 95% percent uncertainty intervals representing the 2·5th and 97·5th percentiles of the 1,000 sampled trajectories. The impact of both interventions was defined as the cumulative number of incident

and prevalent TB cases and TB deaths that was averted in the population (compared to the baseline scenario of no targeted interventions) during a 10-year period (2016 - 2025).

S7. Posterior estimates for the natural history of TB by history of TB treatment

Posterior estimates for parameters describing the natural history of TB among treatment-experienced and treatment-naïve people are shown in Figures S3-S6.

S8. Sensitivity and scenario analyses

Detailed results for the sensitivity analysis as described in the main document are shown in Table S18 and Figure S7(A). Results from additional scenario analyses are illustrated in Figures S7(B und C), S8 and S9.

Table S18: Sensitivity analysis: Partial Rank Correlation Coefficients (PRCC)

Model parameter	PRCC	P-Value
**Demographics**		
Annual per capita birth rate	-0·042	0·176
Annual natural death rate among children (<15 years)	-0·126	<0·001
Annual natural death rate among adults (≥15 years)	0·001	0·977
Natural death rate ratio, TB treatment-experienced adults to treatment-naïve adults	-0·459	<0·001
**Probability of Fast Progression to Active TB Upon Primary Infection**		
Adults, susceptible/treatment-naïve/HIV-	-0·273	<0·001
Adults, susceptible/treatment-naïve/HIV+/non-immunocompromised	0·033	0·305
Adults, susceptible/treatment-naïve/HIV+/immunocompromised	-0·064	0·045
Children, susceptible	0·043	0·175
**Rate of Reactivation of latent TB infection**		
Adults, latently infected/treatment-naïve/HIV-	-0·220	<0·001
Adults, latently infected/treatment-naïve/HIV+/non-immunocompromised	-0·109	0·001
Adults, latently infected/treatment-naïve/HIV+/immunocompromised	0·088	0·006
Children, latently infected	0·011	0·739
**Percent Reduction in Susceptibility due to Partial Immunity afforded by Prior Infection (treatment-naïve)**		
Adults, latently infected/HIV-	0·277	<0·001
Adults, latently infected/HIV+/non-immunocompromised	0·076	0·016
Adults, latently infected/HIV+/immunocompromised	0·002	0·945
**Rate of Natural Recovery among Undetected Active TB Cases**		
Adults, infectious/treatment-naïve/HIV-	0·036	0·258
Adults, infectious/treatment-naïve/HIV+/non-immunocompromised	-0·035	0·269
Adults, infectious/ prior complete treatment/HIV-	-0·082	0·010
Adults, infectious/prior complete treatment/HIV+/immunocompromised	-0·032	0·315
Adults, infectious/ prior incomplete treatment/HIV-	0·107	0·001
Adults, infectious/prior incomplete treatment/HIV+/immunocompromised	-0·035	0·269
Children, infectious	-0·216	<0·001
**Percent Reduction in Susceptibility due to Partial immunity after (treated) active TB**		
Adults, latently infected/treatment-experienced/HIV-	-0·041	0·200
Adults, latently infected/ treatment-experienced/HIV+/non-immunocompromised	0·115	<0·001
Adults, latently infected/ treatment-experienced/HIV+/immunocompromised	-0·076	0·017
Adults, latently infected/ prior complete or incomplete treatment/HIV+/ART	-0·059	0·064

**Rate of Reactivation of active TB after treatment**		
Adults, prior complete treatment/HIV-	0·507	<0·001
Adults, prior incomplete treatment/HIV-	0·114	<0·001
Adults, prior complete treatment /HIV+/non-immunocompromised	0·008	0·805
Adults, prior incomplete treatment /HIV+/non-immunocompromised	0·121	<0·001
Adults, prior complete treatment/HIV+/ immunocompromised	-0·112	<0·001
Adults, susceptible/ prior incomplete treatment /HIV+/immunocompromised	0·134	<0·001
**Probability of Persistent Active TB Following Incomplete Treatment**		
Adults, prior incomplete treatment/HIV-	-0·061	0·055
Adults, prior incomplete treatment/HIV+/non-immunocompromised	0·130	<0·001
Adults, prior incomplete treatment/ HIV+/immunocompromised	0·108	0·001
Adults, prior incomplete treatment/ HIV+/ART	0·092	0·004
**Baseline time between disease onset and detection (years)**		
Adults, infectious/treatment-naïve/HIV-	0·018	0·575
Adults, infectious/treatment-naïve/HIV+/non-immunocompromised	0·227	<0·001
Adults, infectious/treatment-naïve/HIV+/immunocompromised	0·065	0·041
Adults, infectious/treatment-naïve/HIV+/ART	-0·064	0·043
Adults, infectious/prior complete treatment/HIV-	0·508	<0·001
Adults, infectious/prior complete treatment /HIV+/non-immunocompromised	-0·233	<0·001
Adults, infectious/prior complete treatment /HIV+/immunocompromised	-0·021	0·502
Adults, infectious/prior complete treatment /HIV+/ ART	0·118	<0·001
Adults, infectious/prior incomplete treatment/HIV-	-0·030	0·350
Adults, infectious/prior incomplete treatment /HIV+/non-immunocompromised	-0·011	0·729
Adults, infectious/prior incomplete treatment /HIV+/immunocompromised	0·068	0·033
Adults, infectious/prior incomplete treatment /HIV+/ ART	0·038	0·232
Children, infectious	0·104	0·001
**Rate of TB-associated (excess) mortality rate, untreated TB**		
Adults, infectious/any or no treatment history/HIV-	0·093	0·003
Adults, infectious/any or no treatment history/HIV+/non-immunocompromised	-0·090	0·004
Adults, infectious/any or no treatment history/HIV+/ immunocompromised	0·254	<0·001
Adults, infectious/any or no treatment history/HIV+/ART	-0·085	0·007
**Rate of TB-associated (excess) mortality rate, on TB treatment**		
Adults, infectious/any or no treatment history/HIV-	0·028	0·376
Adults, infectious/any or no treatment history/HIV+/non-immunocompromised	0·018	0·560
Adults, infectious/any or no treatment history/HIV+/immunocompromised	0·374	<0·001
Adults, infectious/any or no treatment history /HIV+/ART	-0·131	<0·001
**HIV-progression, HIV-associated mortality and effect of ART**		
Annual rate of progression to immunocompromised HIV from non-immunocompromised HIV	0·102	0·001
Annual non-immunocompromised HIV-associated excess mortality rate	0·035	0·270
Annual immunocompromised HIV-associated excess mortality rate	0·095	0·003
Annual HIV-associated excess mortality rate while on ART	0·223	<0·001
Effectiveness of ART in reversing effect of HIV on TB natural history (compared to the HIV+/non-immunocompromised state, excluding mortality)	-0·061	0·054
**Efficacy of 2°IPT**		
Reduction in TB reactivation rate	0·280	<0·001
Reduction in probability of fast progression to TB after reinfection	0·019	0·558
**Susceptibility to infection**		
Ratio: susceptible Children to HIV-negative, susceptible adults	0·358	<0·001
Ratio: latently infected children to HIV-negative, susceptible adults	0·143	<0·001
**Infectiousness**		
Adults, treatment-naïve, HIV-	-0·339	<0·001
Adults, treatment-naïve, HIV+/non-immunocompromised	0·109	0·001
Ratio: adults, HIV+/immunocompromised to adults, HIV+/non-immunocompromised	-0·003	0·926
Ratio: adults, HIV+/on ART to adults, HIV+/non-immunocompromised	0·050	0·117
Ratio: children to treatment-naïve adults	-0·087	0·006
Ratio: adults, treatment-experienced to adults, treatment-naïve	0·043	0·172

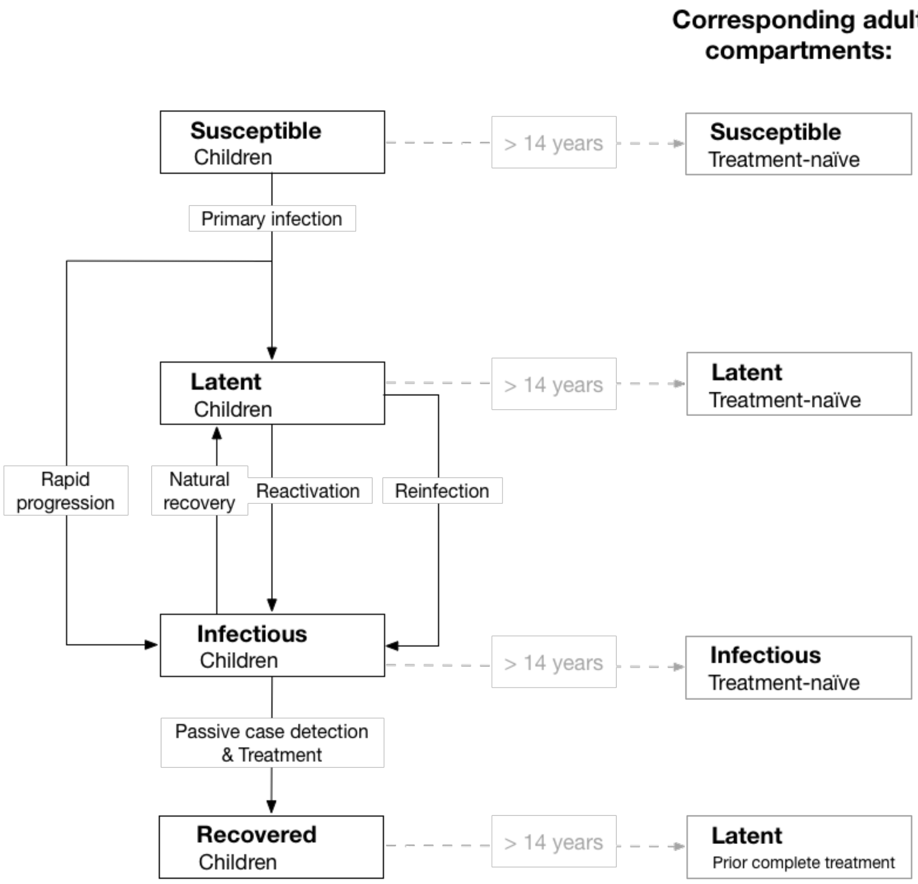

Figure S1: Model subcomponent for children aged 0-14 years
Not shown are mortality rates; grey dashed arrows indicate age transition into the corresponding compartments of the adult component of the model (see Figure 1, main manuscript)

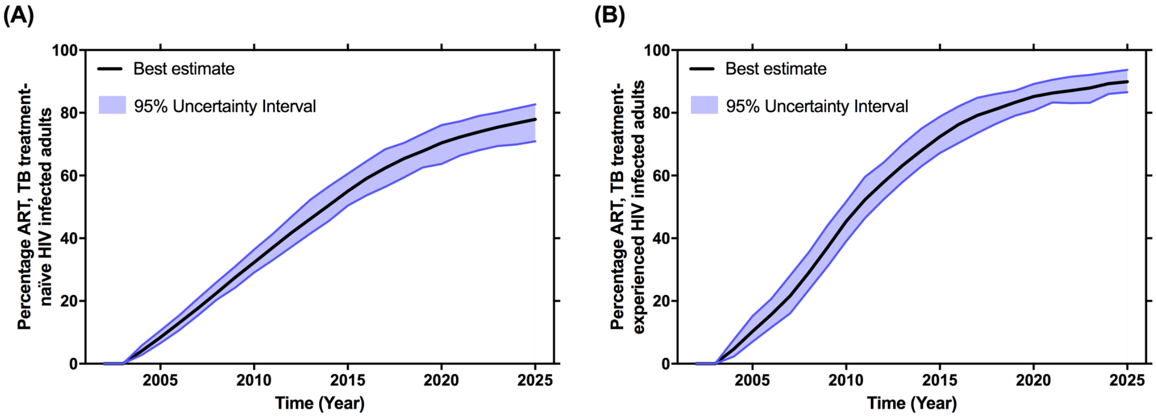

Figure S2. Projected coverage of antiretroviral treatment (ART) among HIV infected adults, 2004 - 2025
Panel A: treatment-naive adults
Panel B: treatment-experienced adults

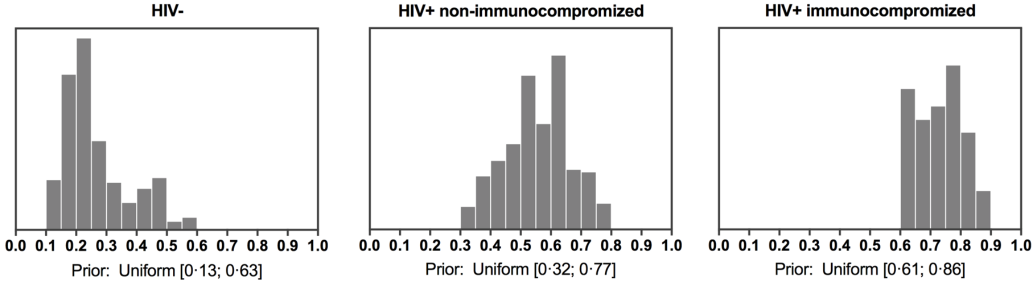

Figure S3: Posterior distribution for the relative susceptibility to reinfection among treatment-naïve, latently infected adults using the susceptibility to primary infection among treatment-naïve, susceptible adults as a reference (assuming partial immunity afforded by prior infection)

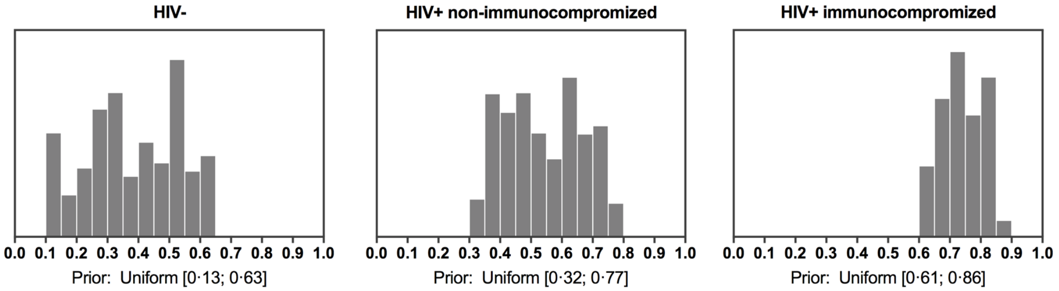

Figure S4: Posterior distribution for the relative susceptibility to reinfection among treatment-experienced adults using the susceptibility to primary infection among treatment-naïve, susceptible adults as a reference (assuming partial immunity afforded by prior infection)

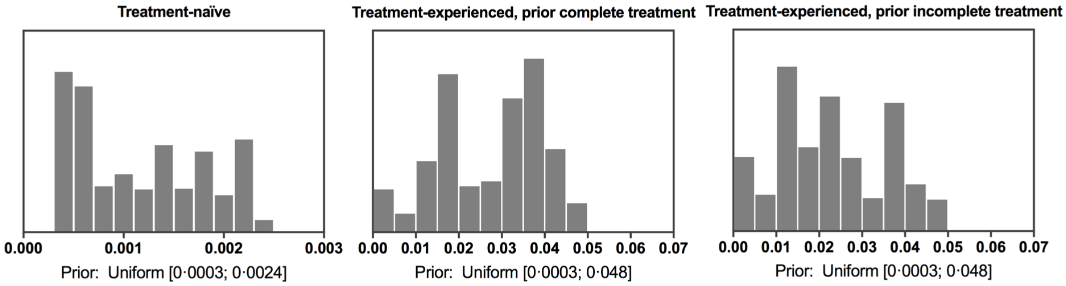

Figure S5: Posterior distribution for the annual reactivation rate among HIV-negative latently-infected adults, by history of previous TB treatment

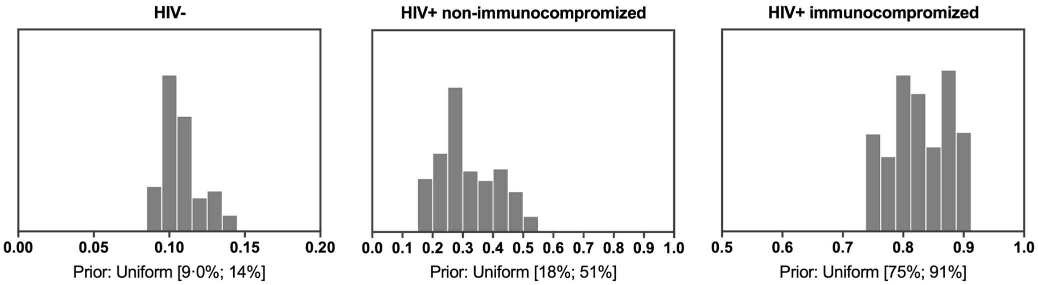

Figure S6: Posterior distribution for the probability of fast progression to active TB upon primary infection by status of HIV co-infection, treatment-naïve, susceptible adults

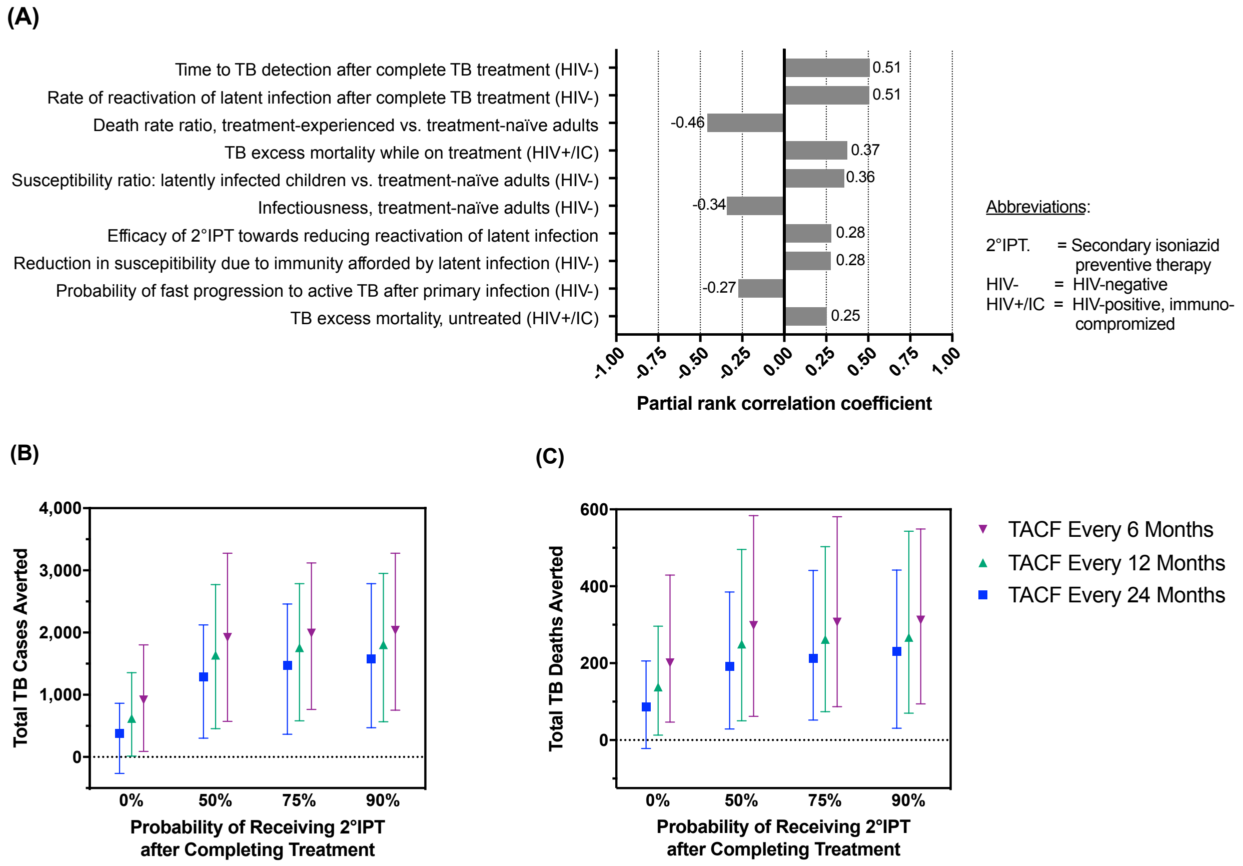

Figure S7: Sensitivity and scenario analyses: Partial rank correlation coefficients for the top 10 model parameters with the greatest influence towards the number of TB cases averted through TACF and 2°IPT interventions (5A); expected number of TB cases averted (5B) and deaths averted (5C) as the result of TACF and 2°IPT interventions with respect to the baseline scenario for varying TACF intervals and probabilities of enrollment in 2°IPT after the completion of TB treatment. Note that the space between data points for different series (5B/5C) is intended to improve readability and is not proportional to scale of the x-axis; error bars represent 95% uncertainty intervals.

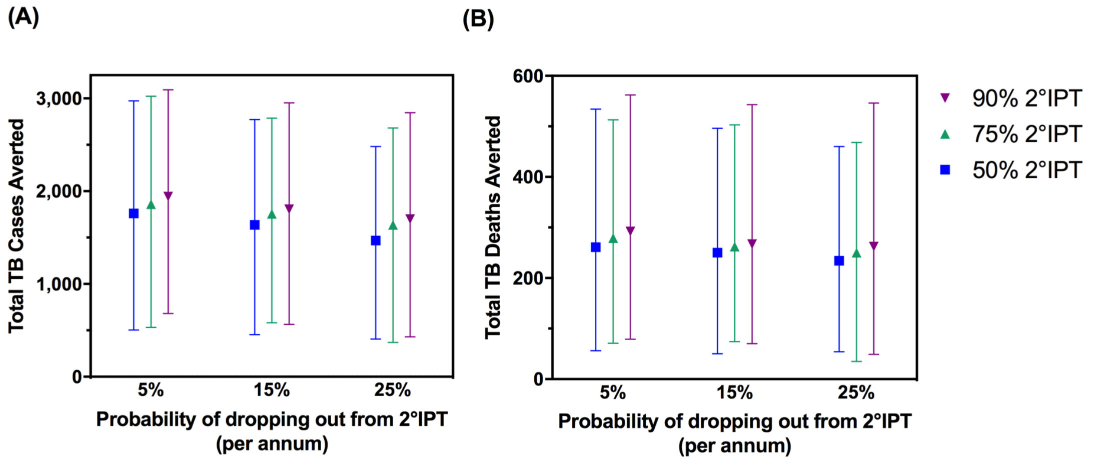

Figure S8: Expected number of TB cases (Fig. A) and deaths (Fig. B) averted with respect to the baseline scenario as the result of annual TACF and 2°IPT when the probability of annual 2°IPT drop-out varied between 5% and 25%. Series represent different probabilities of receiving 2°IPT after completing TB treatment (50%-90%; see legend); space between data points of different series is for better readability and not proportional to scale of the x-axis; error bars represent 95% uncertainty intervals.

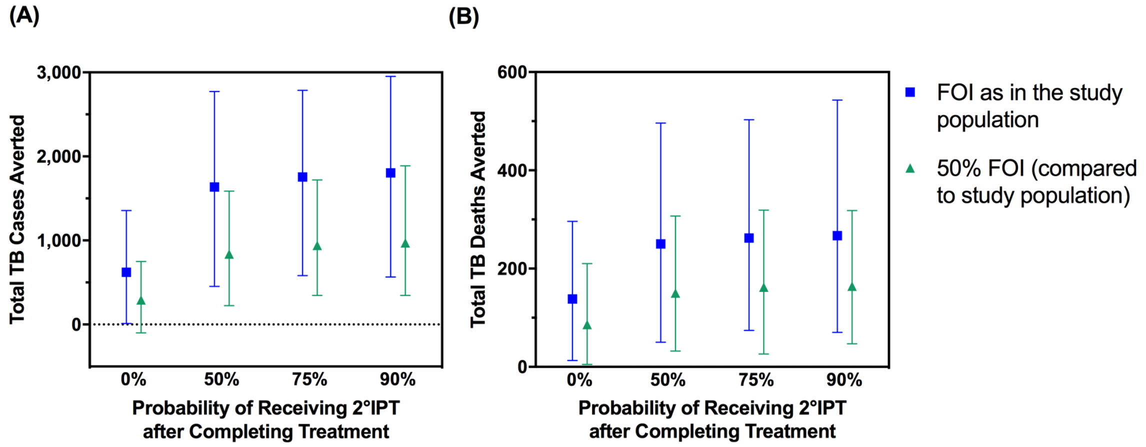

Figure S9: Expected number of TB cases (Fig. A) and deaths (Fig. B) averted as the result of annual TACF and 2°IPT interventions with respect to a scenario where the TB force-of-infection (FOI) is reduced by 50% compared to the TB force-of-infection estimated for our study population. Space between data points of different series is for better readability and not proportional to scale of the x-axis; error bars represent 95% uncertainty intervals.

Appendix References

1. Munch Z, Van Lill SW, Booysen CN, Zietsman HL, Enarson DA, Beyers N. Tuberculosis transmission patterns in a high-incidence area: a spatial analysis. *Int J Tuberc Lung Dis* 2003; **7**(3): 271-7.
2. Kritzinger FE, den Boon S, Verver S, et al. No decrease in annual risk of tuberculosis infection in endemic area in Cape Town, South Africa. *Trop Med Int Health* 2009; **14**(2): 136-42.
3. Verver S, Warren RM, Munch Z, et al. Transmission of tuberculosis in a high incidence urban community in South Africa. *Int J Epidemiol* 2004; **33**(2): 351-7.
4. van Rie A, Warren R, Richardson M, et al. Exogenous reinfection as a cause of recurrent tuberculosis after curative treatment. *N Engl J Med* 1999; **341**(16): 1174-9.
5. Verver S, Warren RM, Beyers N, et al. Rate of reinfection tuberculosis after successful treatment is higher than rate of new tuberculosis. *Am J Respir Crit Care Med* 2005; **171**(12): 1430-5.
6. Marx FM, Dunbar R, Enarson DA, et al. The temporal dynamics of relapse and reinfection tuberculosis after successful treatment: a retrospective cohort study. *Clin Infect Dis* 2014; **58**(12): 1676-83.
7. Marx FM, Dunbar R, Enarson DA, Beyers N. The rate of sputum smear-positive tuberculosis after treatment default in a high-burden setting: a retrospective cohort study. *PLoS One* 2012; **7**(9): e45724.
8. den Boon S, van Lill SW, Borgdorff MW, et al. High prevalence of tuberculosis in previously treated patients, Cape Town, South Africa. *Emerg Infect Dis* 2007; **13**(8): 1189-94.
9. City of Cape Town: 2003 - 2006 - Health Indicators - Tygerberg Sub-District; available at: http://www.capetown.gov.za/EN/CITYHEALTH/HEALTHINFORMATION/Pages/TygerbergSub-District.aspx (Accessed: 14th December 2017).
10. Waitt CJ, Squire SB. A systematic review of risk factors for death in adults during and after tuberculosis treatment. *Int J Tuberc Lung Dis* 2011; **15**(7): 871-85.
11. Shuldiner J, Leventhal A, Chemtob D, Mor Z. Mortality of tuberculosis patients during treatment in Israel, 2000-2010. *Int J Tuberc Lung Dis* 2014; **18**(7): 818-23.
12. Miller TL, Wilson FA, Pang JW, et al. Mortality hazard and survival after tuberculosis treatment. *Am J Public Health* 2015; **105**(5): 930-7.
13. Buist AS, McBurnie MA, Vollmer WM, et al. International variation in the prevalence of COPD (the BOLD Study): a population-based prevalence study. *Lancet* 2007; **370**(9589): 741-50.
14. Tocque K, Convrey RP, Bellis MA, Beeching NJ, Davies PD. Elevated mortality following diagnosis with a treatable disease: tuberculosis. *Int J Tuberc Lung Dis* 2005; **9**(7): 797-802.
15. Mossong J, Hens N, Jit M, et al. Social contacts and mixing patterns relevant to the spread of infectious diseases. *PLoS Med* 2008; **5**(3): e74.
16. Daley CL, Small PM, Schecter GF, et al. An outbreak of tuberculosis with accelerated progression among persons infected with the human immunodeficiency virus. An analysis using restriction-fragment-length polymorphisms. *N Engl J Med* 1992; **326**(4): 231-5.
17. Di Perri G, Cruciani M, Danzi MC, et al. Nosocomial epidemic of active tuberculosis among HIV-infected patients. *Lancet* 1989; **2**(8678-8679): 1502-4.
18. Selwyn PA, Hartel D, Lewis VA, et al. A prospective study of the risk of tuberculosis among intravenous drug users with human immunodeficiency virus infection. *N Engl J Med* 1989; **320**(9): 545-50.
19. Kunkel A, Abel Zur Wiesch P, Nathavitharana RR, Marx FM, Jenkins HE, Cohen T. Smear positivity in paediatric and adult tuberculosis: systematic review and meta-analysis. *BMC Infect Dis* 2016; **16**(1): 282.
20. Vynnycky E, Fine PE. The natural history of tuberculosis: the implications of age-dependent risks of disease and the role of reinfection. *Epidemiol Infect* 1997; **119**: 183-201.
21. Dye C, Garnett GP, Sleeman K, Williams BG. Prospects for worldwide tuberculosis control under the WHO DOTS strategy. Directly observed short-course therapy. *Lancet* 1998; **352**: 1886-91.
22. Menzies NA, Cohen T, Lin HH, Murray M, Salomon JA. Population health impact and cost-effectiveness of tuberculosis diagnosis with Xpert MTB/RIF: a dynamic simulation and economic evaluation. *PLoS Med* 2012; **9**: e1001347.
23. Marais BJ, Gie RP, Schaaf HS, et al. The clinical epidemiology of childhood pulmonary tuberculosis: a critical review of literature from the pre-chemotherapy era. *Int J Tuberc Lung Dis* 2004; **8**(3): 278-85.
24. Dowdy DW, Chaisson RE. The persistence of tuberculosis in the age of DOTS: reassessing the effect of case detection. *Bull World Health Organ* 2009; **87**: 296-304.

25. Ferebee SH. Controlled chemoprophylaxis trials in tuberculosis. A general review. *Bibliotheca tuberculosea* 1970; **26**: 28-106.
26. Dye C, Williams BG. Criteria for the control of drug-resistant tuberculosis. *Proc Natl Acad Sci U S A* 2000; **97**(14): 8180-5.
27. Dye C, Espinal MA. Will tuberculosis become resistant to all antibiotics? *Proc Biol Sci* 2002; **268**: 45-52.
28. Cohen T, Lipsitch M, Walensky RP, Murray M. Beneficial and perverse effects of isoniazid preventive therapy for latent tuberculosis infection in HIV-tuberculosis coinfected populations. *Proc Natl Acad Sci U S A* 2006; **103**: 7042-7.
29. Datiko DG, Lindtjorn B. Cost and cost-effectiveness of smear-positive tuberculosis treatment by Health Extension Workers in Southern Ethiopia: a community randomized trial. *PLoS One* 2010; **5**(2): e9158.
30. Marx FM, Floyd S, Ayles H, Godfrey-Faussett P, Beyers N, Cohen T. High burden of prevalent tuberculosis among previously treated people in Southern Africa suggests potential for targeted control interventions. *The European respiratory journal : official journal of the European Society for Clinical Respiratory Physiology* 2016; **48**(4): 1227-30.
31. Corbett EL, Charalambous S, Moloi VM, et al. Human immunodeficiency virus and the prevalence of undiagnosed tuberculosis in African gold miners. *Am J Respir Crit Care Med* 2004; **170**(6): 673-9.
32. Corbett EL, Bandason T, Cheung YB, et al. Epidemiology of tuberculosis in a high HIV prevalence population provided with enhanced diagnosis of symptomatic disease. *PLoS Med* 2007; **4**(1): e22.
33. Manosuthi W, Tantanathip P, Chimsuntorn S, et al. Treatment outcomes of patients co-infected with HIV and tuberculosis who received a nevirapine-based antiretroviral regimen: a four-year prospective study. *International journal of infectious diseases : IJID : official publication of the International Society for Infectious Diseases* 2010; **14**(11): e1013-7.
34. van der Sande MA, Schim van der Loeff MF, Bennett RC, et al. Incidence of tuberculosis and survival after its diagnosis in patients infected with HIV-1 and HIV-2. *AIDS* 2004; **18**(14): 1933-41.
35. Mahy M, Lewden C, Brinkhof MW, et al. Derivation of parameters used in Spectrum for eligibility for antiretroviral therapy and survival on antiretroviral therapy. *Sex Transm Infect* 2010; **86 Suppl 2**: ii28-34.
36. Todd J, Glynn JR, Marston M, et al. Time from HIV seroconversion to death: a collaborative analysis of eight studies in six low and middle-income countries before highly active antiretroviral therapy. *Aids* 2007; **21 Suppl 6**: S55-63.
37. Anglaret X, Minga A, Gabillard D, et al. AIDS and non-AIDS morbidity and mortality across the spectrum of CD4 cell counts in HIV-infected adults before starting antiretroviral therapy in Cote d'Ivoire. *Clin Infect Dis* 2012; **54**(5): 714-23.
38. Badri M, Lawn SD, Wood R. Short-term risk of AIDS or death in people infected with HIV-1 before antiretroviral therapy in South Africa: a longitudinal study. *Lancet* 2006; **368**(9543): 1254-9.
39. Egger M, May M, Chêne G, et al. Prognosis of HIV-1-infected patients starting highly active antiretroviral therapy: a collaborative analysis of prospective studies. *Lancet* 2002; **360**(9327): 119-29.
40. May M, Sterne JAC, Sabin C, et al. Prognosis of HIV-1-infected patients up to 5 years after initiation of HAART: collaborative analysis of prospective studies. *AIDS* 2007; **21**(9): 1185-97.
41. Phillips A, Pezzotti P, Collaboration C. Short-term risk of AIDS according to current CD4 cell count and viral load in antiretroviral drug-naive individuals and those treated in the monotherapy era. *AIDS* 2004; **18**(1): 51-8.
42. Suthar AB, Lawn SD, del Amo J, et al. Antiretroviral therapy for prevention of tuberculosis in adults with HIV: a systematic review and meta-analysis. *PLoS Med* 2012; **9**(7): e1001270.
43. National Tuberculosis Management Guidelines; Department: Health, Republic of South Africa, 2008. Available from: http://www.who.int/hiv/pub/national_guidelines/en (Accessed: 14th December 2017).
44. Annual Report. Metropole District Health Services, Cape Town, 2003-2004. Available from: https://www.westerncape.gov.za/text/2005/2/mdhs_part1.pdf (Accessed: 14th December 2017).
45. Menzies NA, Soeteman DI, Pandya A, Kim JJ. Bayesian Methods for Calibrating Health Policy Models: A Tutorial. *Pharmacoeconomics* 2017; **35**(6): 613-24.
